# Dual Effects of Maternal Diet and Perinatal Organophosphate Flame Retardant Treatment on Offspring Development, Behavior and Metabolism

**DOI:** 10.3390/toxics13080639

**Published:** 2025-07-29

**Authors:** Ali Yasrebi, Catherine M. Rojas, Shabree Anthony, Samantha Feltri, Jamilah Evelyn, Kimberly Wiersielis, Samantha Adams, Veronia Basaly, Grace L. Guo, Lauren M. Aleksunes, Troy A. Roepke

**Affiliations:** 1 Department of Animal Sciences, School of Environmental and Biological Sciences, Rutgers, The State University of New Jersey, New Brunswick, NJ 08901, USAjce87@scarletmail.rutgers.edu (J.E.);; 2 Joint Graduate Program in Toxicology, Rutgers, The State University of New Jersey, Piscataway, NJ 08854, USAsa1558@gsbs.rutgers.edu (S.A.); vb384@scarletmail.rutgers.edu (V.B.);; 3 Department of Biobehavioral Health, College of Health and Human Development, Pennsylvania State University, University Park, PA 16802, USA; 4 Department of Pharmacology and Toxicology, Ernest Mario School of Pharmacy, Rutgers, The State University of New Jersey, Piscataway, NJ 08854, USA; 5 Environmental and Occupational Health Sciences Institute, Rutgers, The State University of New Jersey, Piscataway, NJ 08854, USA

**Keywords:** organophosphate flame retardants, endocrine disruption, neurodevelopment, anxiety, memory

## Abstract

The maternal–fetal environment is influenced by multiple factors, including nutrition and environmental contaminants, which can impact long-term development. Perinatal exposure to organophosphate flame retardants (OPFRs) disrupts energy homeostasis and causes maladaptive behaviors in mice. Maternal obesity affects development by impairing blood–brain barrier (BBB) formation, influencing brain regions involved in energy regulation and behavior. This study examined the combined effects of maternal obesity and perinatal OPFR treatment on offspring development. Female mice were fed either a low-fat (LFD) or a high-fat diet (HFD) for 8 weeks, mated, and treated with either sesame oil or an OPFR mixture (tris(1,3-dichloro-2-propyl)phosphate, tricresyl phosphate, and triphenyl phosphate, 1 mg/kg each) from gestational day 7 to postnatal day 14. Results showed that both maternal diet and OPFR treatment disrupted blood–brain barrier integrity, energy balance, and reproductive gene expression in the hypothalamus of neonates. The expression of hepatic genes related to lipid and xenobiotic metabolism was also altered. In adulthood, LFD OPFR-treated female offspring exhibited increased avoidance behavior, while HFD OPFR-treated females demonstrated memory impairments. Metabolic assessments revealed decreased energy expenditure and nighttime activity in LFD OPFR-treated females. These findings suggest that maternal diet and OPFR treatment alter hypothalamic and liver gene expression in neonates, potentially leading to long-term metabolic and behavioral changes.

## 1. Introduction

Flame retardants have been widely used since the 1970s to prevent the expansion of fires. Although polybrominated diphenyl ethers (PBDEs) were phased out following global bans, organophosphate flame retardants (OPFRs) have emerged as a “regrettable substitution”. OPFRs are now commonly found in both the environment and indoor settings, such as homes and offices. Because these compounds are not covalently bound to everyday items—such as furniture, desks, or mattresses—they accumulate in dust, leading to various routes of human exposure, including ingestion, inhalation, dermal absorption, and in utero transfer [1,2,3,4,5,6]. In 2018, the estimated OPFR daily intake through inhalation, dermal absorption, and dust ingestion was 149, 279, and 390 ng/kg bw/day, respectively [7].

OPFRs have been negatively associated with harmful health outcomes in humans, including effects on birth weight, thyroid hormone regulation, placental function, and neurodevelopment [8,9,10,11,12]. Animal studies have elucidated potential mechanisms underlying these outcomes. Our previous work has shown that both perinatal [13] or adult [14] exposure to tris(1,3-dichloro-2-propyl)phosphate (TDCPP), tricresyl phosphate (TCP), and triphenyl phosphate (TPP) disrupts energy homeostasis by altering gene expression in the hypothalamus and liver, primarily through nuclear hormone receptors (NHRs)/transcription factors, such as peroxisome proliferator-activated receptor gamma (PPARγ) and estrogen receptor alpha (ERα). Numerous studies, including our own, have reported similar effects. For example, the Conditions Affecting Neurocognitive Development and Learning in Early Childhood (CANDLE) study recently demonstrated that OPFRs disrupt functions of NHRs in the placenta, including PPARγ and retinoic acid receptors (RARs) [10]. Dysregulation of NHR expression and activity by flame retardants has been associated with altered lipid metabolism, ultimately impairing energy homeostasis [15,16,17,18,19,20,21]. Collectively, these data strengthen the evidence that OPFRs function as environmental obesogens through endocrine-disruptive pathways.

In 2019, 29% of women in the United States were obese prior to becoming pregnant, an 11% increase from 2016, which continues the upward trend in previous reports [22,23,24]. Maternal obesity is associated with a wide range of adverse pregnancy outcomes, including gestational hypertension and diabetes, preeclampsia, preterm birth, and an elevated risk of miscarriage or congenital defects [25]. Moreover, studies have shown that in utero exposures—such as maternal high-fat diet and environmental obesogens—can increase susceptibility to metabolic syndrome [26,27]. Maternal obesity has been shown to impair fetal and neonatal developmental trajectories by altering epigenetic programming [28]. It also increases the permeability of the neonatal blood–brain barrier, which ultimately impacts neurodevelopment [29,30].

We aim to investigate how perinatal OPFR treatment and maternal obesity influence the development of offspring during both neonatal and adult stages. Data from the National Health and Nutrition Examination Survey (NHANES) have identified associations between organophosphate ester (OPE) exposure and increased body mass index (BMI) and blood pressure across children, adolescents, and adults [31,32]. However, findings regarding the relationship between gestational OPE exposure and childhood obesity have been mixed. For instance, exposure to di-isobutyl phosphate (DBUP) and di-isobutyl phthalate (DIBP) has been linked to increased obesity risk, whereas bis(1,3-dichloro-2-propyl)phosphate (BDCPP) exposure was inversely associated with obesity [33]. In contrast, prior epidemiological studies on brominated flame retardants have shown that maternal exposure predisposes male offspring to elevated BMI in childhood [34].

Our laboratory has established a maternal OPFR exposure model based on environmentally relevant concentrations of these compounds, reflecting real-world mixtures that interact with ERα [13,14]. Using this model, our previous studies have shown that maternal OPFR exposure programs peripheral organ function in a sex-dependent manner, influencing offspring responses to an obesogenic diet. In adult male mice, these effects include altered substrate utilization, energy expenditure, feeding behavior, and elevated blood pressure [35,36].

Additionally, there are numerous studies demonstrating that maternal obesity or high-fat diet (HFD) intake alters neonatal and juvenile brain development, leading to the disruption of energy homeostasis in adult offspring [37,38,39,40,41]. We have shown that the absence of ERα blocks the effects of maternal HFD on offspring energy homeostasis. Conversely, partial restoration of estrogen response element (ERE)-independent ERα signaling reinstates susceptibility to maternal HFD, potentially through epigenetic regulation of ERα in the developing brain [42]. Furthermore, maternal exposure to a 45% HFD or a diet high in linoleic acid exacerbates the effects of an adult offspring HFD, particularly by impairing glucose metabolism [43,44].

Given these findings, it is critical to understand the potential synergistic effects of maternal diet and OPFR treatment during development on offspring gene expression, metabolism, physiology, and behavior. The objective of the present study is to evaluate the impact of these two factors, which are known to disrupt energy homeostasis and neurodevelopment. We assessed changes after in utero (PND 0) and lactational OPFR exposures (PND 14) related to gene expression in the hypothalamus and the liver, since these tissues are particularly sensitive to developmental EDC exposures [45]. Our results revealed sex- and maternal diet-specific differences in the metabolism and behavior of adult offspring. Collectively, our findings suggest that maternal obesity and perinatal OPFR treatment alter hypothalamic and hepatic gene expression in a sex-dependent manner, which may underlie the observed alterations in adult physiology, metabolism, and behavior.

## 2. Materials and Methods

### 2.1. Animal Care and Experimental Design

All animal treatments and procedures were completed in accordance with National Institutes of Health standards and Rutgers Institutional Animal Care and Use Committee and adhered to ARRIVE’s guidelines for reporting animal research. All wild-type (WT) C57Bl/6J (Jackson Laboratory, Bar Harbor, ME, USA) female and male mice were bred in-house and maintained under controlled temperature (25 °C), humidity (30–70%), and a 12 h/12 h light/dark cycle with ad libitum access to water and food. Female unmated mice were fed a low-fat diet (LFD; 10% kCal fat, Cat D12450H, Research Diets, New Brunswick, NJ, USA) or high-fat diet (HFD; 45% kCal fat, Cat D12451, Research Diets) for 8 weeks prior to mating until dam sacrifice. All females were weighed weekly, and their body composition using EchoMRI 3-in-1 Body Composition Analyzer (Echo Medical Systems, Houston, TX, USA), and fasting glucose levels were measured 1 week prior to mating. Females were pair housed with males for 1 week.

**Figure 1:**
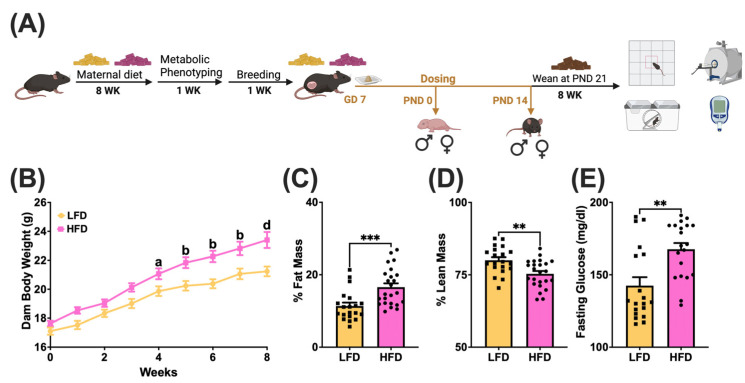
(**A**) Schematic of experimental design. GD, gestational day; PND, postnatal day. Dams were fed diets from 6 weeks of age until sacrifice. Metabolic phenotyping was completed after 8 weeks of diet, including (**B**) body weight (a = *p* < 0.05, b = *p* < 0.01, d = *p* < 0.0001), (**C**) fat mass, and (**D**) lean mass normalized to body mass. (**E**) Five-hour fasting glucose levels were measured after 8 weeks of diet intervention using a standard glucometer (** = *p* < 0.01, *** = *p* < 0.001).

Gestational day (GD) 0 was determined by confirmation of a vaginal plug. Dams were weighed every 3 days until GD 16, then every 2 days thereafter to adjust dose concentrations accordingly. Both LFD and HFD dams had consistent weight gain, increasing ~3 g every 3 days after GD 7 and ~2 g every 2 days after GD 16. Dams received a daily oral dose from GD 7 to postnatal day (PND) 14 of 1 mg/kg body weight. Dams were randomly assigned to the following treatment groups: sesame oil vehicle combined with powdered peanut butter (PB2^®^, PB2 Foods, Tifton, GA, USA) or OPFR mixture and sesame oil combined with powdered peanut butter. One male pup and one female pup were euthanized at PND 0 and 14 for whole brain and liver collection. On PND 7, the anogenital distance (AGD), was measured with calipers in all litters. Remaining pups from each litter were weaned at PND 21 and fed a standard, low-phytoestrogen chow diet (Lab Diets, 5V75, Richmond, IN, USA). Offspring were housed and fed ad libitum until 8 weeks of age, at which point behavioral, metabolic, and physiological tests were conducted (Figure 1A). After all tests were conducted, offspring were fasted at 900 h and euthanized at 1000 h by decapitation.

### 2.2. Flame Retardants

The OPFR mixture consisted of tricresyl phosphate (TCP, AccuStandard, New Haven, CT, USA, CAS#:1330-78-5, 99%), tris(1,3-dichloro-2-propyl)phosphate (TDCPP, Sigma Aldrich, St. Louis, MO, USA, CAS#:13674-87-8, 95.6%), and triphenyl phosphate (TPP, Sigma Aldrich, St. Louis, MO, USA; CAS#:115-86-6, 99%). As previously described [13], 100 mg of each OPFR was dissolved in 1 mL of acetone (Sigma-Aldrich, Saint Louis, MO, USA), then 100 µL of the acetone stock solution was added to 10 mL of sesame oil and allowed to vent for 48 h.

### 2.3. Tissue Preparation and Quantitative Real-Time PCR

Mediobasal hypothalamic (MBH) sections were microdissected from PND 0 and PND 14 pups under a dissecting microscope as previously described [13]. MBH was preserved in RNALater (Life Technologies, Grand Island, NY, USA) at −80 °C, and RNA was extracted using RNAqueous-Micro Kits (Life Technologies). The whole liver was submerged in RNALater, stored at −80 °C, and RNA was extracted using a Trizol extraction coupled with a NucleoSpin RNA kit (Macherey-Nagel, Bethlehem, PA, USA). Approximately 20 mg of liver was used for each sample. MBH and liver RNA quantity and quality were determined using a NanoDrop-2000 spectrophotometer (ThermoFisher, Waltham, MA, USA), and RIN was determined using an Agilent Bioanalyzer (only RIN > 7.0 were used). RNA concentrations were standardized prior to reverse transcription and diluted 1:20 for a final concentration of 0.5 ng cDNA for MBH and 1.5 ng cDNA for liver. cDNA was synthesized as previously described [14] and 4 mL of cDNA was amplified using Power SYBR Green (ThermoFisher) or SSO Advanced (BioRad, Hercules, CA, USA) Master Mix. Refer to Supplementary Tables S1 and S2 for the complete list of primer sequences used in these experiments. Relative gene expression was determined using the δδCT method calculated by the geomean of reference genes *Actb*, *Gapdh*, and *Hprt*.

### 2.4. Behavior Assays

Behavioral assays were conducted between 900 and 1200 h using a white light and occurred during the light phase of the light/dark cycle. Adult male offspring were tested before female offspring each day. The chambers were cleaned with MB-10 (Quip labs, Wilmington, DE, USA) spray in between animals to ensure that behavior was not influenced by scent. Mice were acclimated to the behavior room for at least 24 h prior to the first test. The mice were tested on avoidance behaviors in the following order: open field test (OFT), elevated plus maze (EPM), and light/dark box emergence test (LDB). Hippocampal-dependent memory was also assessed using the Y-maze test, which was the last test in the sequence. EPM was conducted second to allow for washout between the OFT and LDB, since they are conducted in the same apparatus, and Y-maze was conducted last to not interfere with avoidance behavior analysis. All tests were recorded and analyzed by the ANY-maze behavior monitoring software (ANY-maze, Version 6, Stoelting, Wood Dale, IL, USA). Vaginal cytology was performed on the female offspring after each behavior test to confirm the estrous stage.

The OFT apparatus measures 40 cm long × 40 cm wide × 40 cm high with an open top and a 64-square grid floor. The mice were placed in the bottom left corner of the arena at the start of the test, and the ANY-maze software measured activity for 10 min. The EPM apparatus has 4 arms that are 30 cm long × 5 cm wide and intersect at the 5 cm square center. The walls that enclose 2 of the arms are 15 cm tall with open tops. The mice were placed into the center of the apparatus at the start of the test, which lasted 5 min. The LDB test is conducted in the OFT apparatus with a black insert that is 20 cm long × 40 cm wide × 40 cm tall. The mice were placed in the bottom left corner of the arena, and the ANY-maze software measured activity for 10 min. The Y-maze apparatus has three identical arms, which measure 8 cm wide × 15 cm high × 30 cm long and are connected to a triangular center. The mice were initially placed in the start arm and allowed to explore this arm and the habituation arm for 5 min while the test arm was closed off using a white opaque removable door. After completion of the habituation phase, mice were held in a temporary cage for a 5 min intertrial delay. After the delay, the mice were returned to the start arm and allowed to freely explore all 3 arms for 5 min. Exclusion criteria for each behavior assay included mice that did not enter all zones at least once, or if the test did not reach full duration, i.e., technical difficulties with software.

### 2.5. Metabolic and Physiological Parameters

Body composition (fat and lean mass) was assessed by Echo MRI™ Body Composition (Houston, TX, USA) for all offspring after behavior tests at 8 weeks of age. The Comprehensive Lab Animal Monitoring System (CLAMS, Columbus Instruments, Columbus, OH, USA) was used to measure oxygen consumption (V.O_2_), carbon dioxide production (V.CO_2_), respiratory exchange ratio (RER), energy expenditure, and general locomotor activity and wheel running under constant 25 °C and 12:12 h light/dark cycle. Mice were single housed and were allowed to acclimate to the chamber for 24 h before the 48 h trial. The general metabolic rate was determined through mouse heat expenditure recordings. Food and water intake, general activity (X, Y, and Z plane), and wheel running were also recorded. Subsequently, all mice underwent glucose and insulin tolerance tests. For the glucose tolerance test (GTT), mice were fasted for 5 h and then intraperitoneally (IP) injected with a bolus of 2 g/kg glucose. Blood glucose was measured from tail bleeds using an AlphaTrak2 glucometer (Zoetis, Parsippany, NJ, USA). Glucose measurements were taken at 0, 15, 30, 60, 90, and 120 min post-injection. One week later, insulin tolerance tests (ITT) were performed using an IP injection of 0.75 U/kg insulin after a 4 h fast. After insulin (Humulin R, Lilly, Indianapolis, IN, USA) injection, glucose was measured in tail blood at 0, 15, 30, 60, 90, and 120 min using a glucometer.

### 2.6. Data Analysis

**Figure 2:**
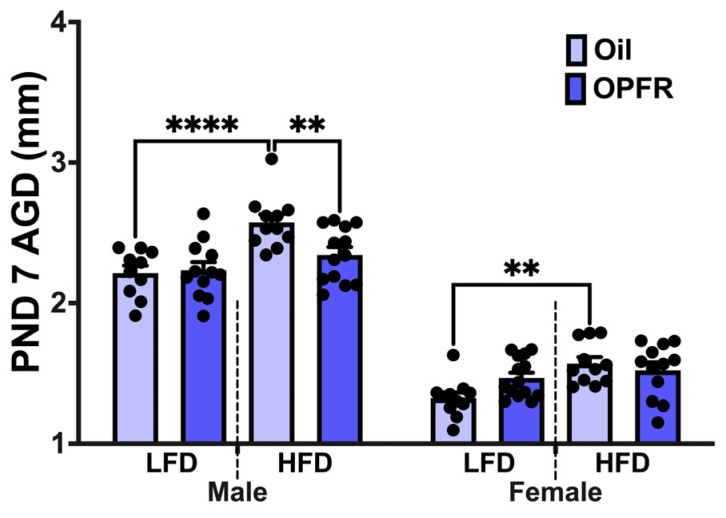
PND 7 anogenital distance (AGD) measurements in male and female pups that were perinatally treated with oil or OPFR and maternally treated with low-fat/high-fat diet (LFD/HFD). Data are represented as mean ± SEM, and dots represent the sample size (number of litters) per treatment per sex (** = *p* < 0.01, **** = *p* < 0.0001).

**Figure 3:**
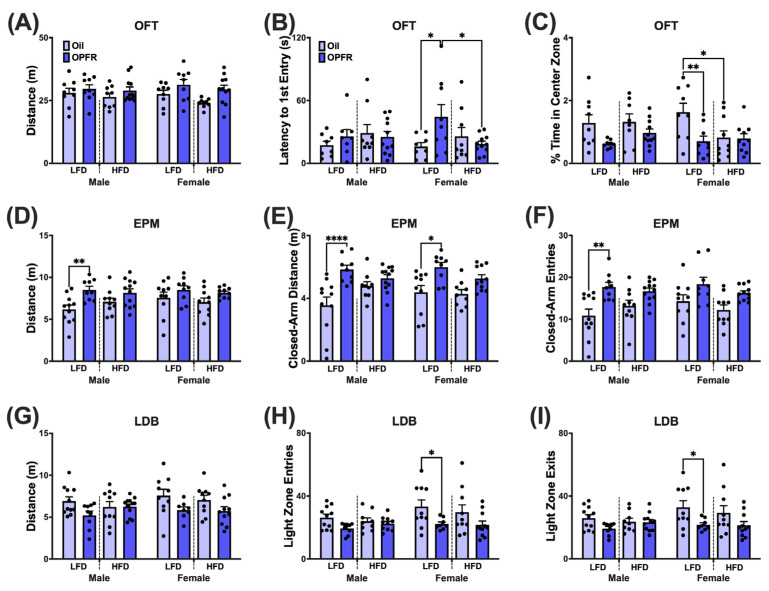
Avoidant-related behavior tests. Open field test (OFT)—(**A**) distance traveled (m), (**B**) latency to 1st entry into the 20 cm center and (**C**) percent time spent in the 10 cm center zone. Elevated plus maze (EPM)—(**D**) distance traveled (m), (**E**) distance traveled (m) in the closed arms, and (**F**) number of closed-arm entries. Light/dark box emergence test (LDB)—(**G**) distance traveled (m), (**H**) number of light zone entries, and (**I**) light zone exits. Data are represented as mean ± SEM and dots represent the sample size (number of litters) per treatment per sex (* = *p* < 0.05, ** = *p* < 0.01, **** = *p* < 0.0001).

**Figure 4:**
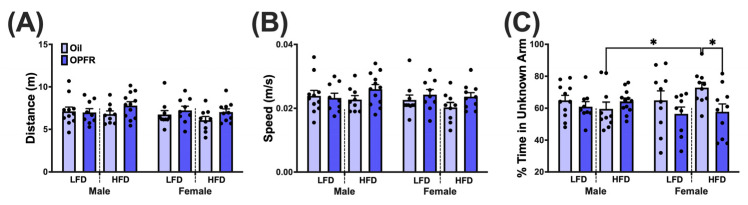
Y-maze Test. (**A**) Distance traveled. (**B**) Mean speed. (**C**) Percent time spent in unknown arm. Data are represented as mean ± SEM and dots represent the sample size (number of litters) per treatment per sex (* = *p* < 0.05).

**Figure 5:**
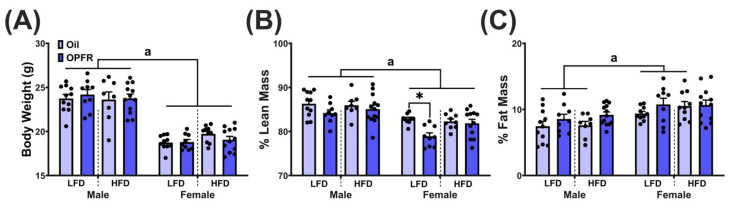
(**A**) Body weights and body composition of maternally treated adult offspring. Lean mass (**B**) and fat mass (**C**) normalized to body weight for adult offspring. Lowercase ‘a’ denotes significance (*p* < 0.05) between sex across treatment groups. Data are represented as mean ± SEM and dots represent the sample size (number of litters) per treatment per sex (* = *p* < 0.05).

**Figure 6:**
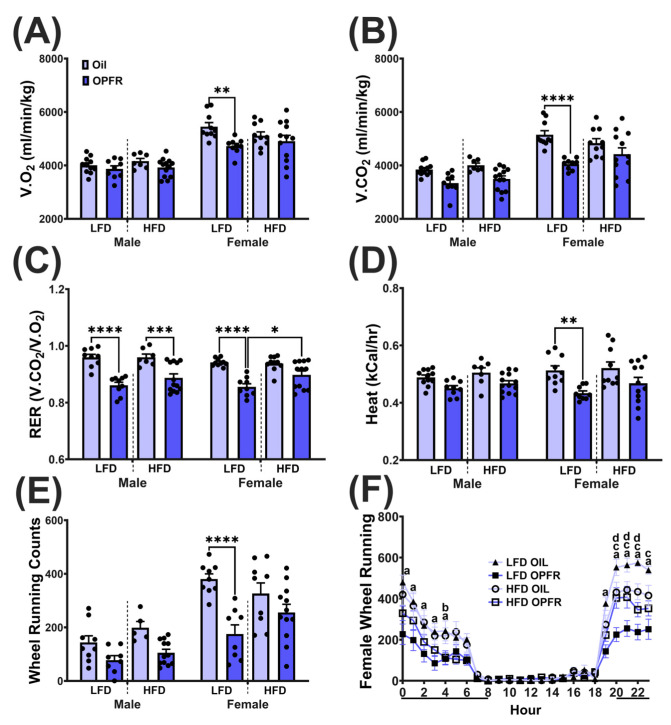
Metabolic phenotyping. Nighttime (**A**) V.O_2_, (**B**) V.CO_2_, (**C**) respiratory exchange ratio, (**D**) Heat, and (**E**) wheel running. Data are presented as mean +/− SEM and analyzed by litter (*n* = 8–12/group) and by two-way ANOVA with Tukey’s multiple comparisons test (* = *p* < 0.05, ** = *p* < 0.01, *** = *p* < 0.001, **** = *p* < 0.0001). (**F**) Female hourly wheel count across 24 h. Dark line below *X*-axis represents dark hours. Letters denote a significance between maternal diet and treatment at each hour (a = *p* < 0.05 for LFD oil and LFD OPFR; b = *p* < 0.05 for HFD oil and HFD OPFR; c = *p* < 0.05 for LFD oil and HFD oil; d = *p* < 0.05 for LFD OPFR and HFD OPFR).

**Figure 7:**
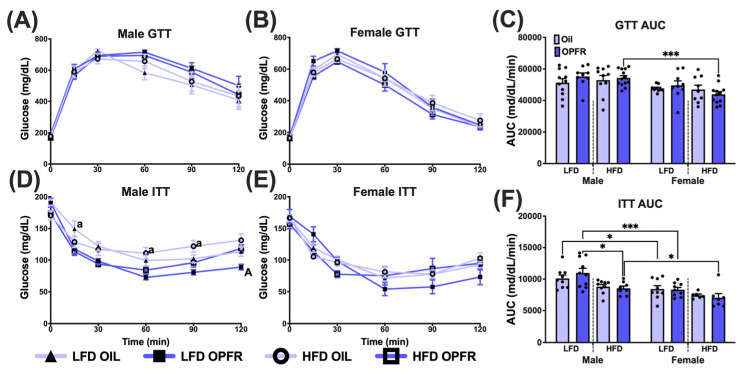
Tolerance tests. Glucose tolerance test (GTT) for (**A**) male and (**B**) female adult offspring. (**C**) Area under the curve (AUC) analysis for adult offspring. Insulin tolerance test (ITT) for (**D**) male and (**E**) female adult offspring. (**F**) AUC analysis for adult offspring. Lowercase letters denote significance between perinatal treatment within maternal diet at the time point, and uppercase letters denote significance between maternal diet within perinatal treatment. Data are represented as mean ± SEM and dots represent the sample size (number of litters) per treatment per sex (A/a = *p* < 0.05, * = *p* < 0.05, *** = *p* < 0.001).

We abided by the ARRIVE Guidelines for animal research. Dam data (Figure 1) were analyzed by 2-way ANOVA for body weight and unpaired *t* tests for fat/lean mass and glucose tests. Offspring data (Figure 2, Figure 3, Figure 4, Figure 5, Figure 6 and Figure 7) were analyzed by litter within each age group by 3-way ANOVA (maternal diet and treatment and sex/time) followed by a post-hoc Tukey test using GraphPad Prism 10 (San Diego, CA, USA), with tests for normality prior to ANOVA. Repeated measures ANOVA was also performed for female hourly wheel running. ANOVA statistics are reported in Supplementary Tables S3–S6. All gene expression data were normalized to LFD oil female pups within PND 0 or PND 14 for comparison of sex, diet, and perinatal treatment. PND 0 and 14 time points were analyzed separately. Results were considered statistically significant at α < 0.05. Bars on graphs represent mean ± SEM of litters.

The outliers for each test were determined using the Grubbs test. In addition, values that exceeded 3 SDs (α < 0.01) above or below the mean were excluded. Fasting glucose was not measured for four HFD-fed dams because of a malfunction in the glucometer. In addition, although 1 male and 1 female from each litter (described in Table 1) were saved for adult studies, some adults had developed cataracts/eye issues (regardless of treatment group), therefore, they were excluded from all tests for consistency. The mice removed due to eye complications are as follows: 1 LFD oil female, 1 LFD OPFR female, 1 HFD OPFR female, and 1 HFD OPFR male. Also, 1 HFD OPFR female was culled due to accidental tail tip removal during GTT and 1 LFD OPFR male was culled due to sickness.

## 3. Results

### 3.1. Maternal Diet-Induced Obesity Model

Dams were fed on LFD or HFD from 6 to 14 weeks of age (Figure 1A). To confirm diet-induced obesity (DIO), dams underwent metabolic phenotyping 1 week prior to breeding. Maternal body weight was recorded from initial feeding until week 8 (Figure 1B). Body composition was analyzed by Echo MRI™, and fasting glucose levels were measured in dams (Figure 1C,D). As expected, body weight (*p* < 0.05) and fat mass (*p* < 0.001) increased while lean mass decreased (*p* < 0.01) in HFD dams. HFD elevated circulating glucose levels (*p* < 0.01) in dams.

### 3.2. Anogenital Distance Measurements at PND 7

In Figure 2, male and female AGD increased after maternal HFD intervention (males: *p* < 0.001; females: *p* < 0.05); however, perinatal OPFR treatment decreased AGD only in male pups within the HFD group (*p* < 0.05).

### 3.3. Blood–Brain Barrier Disruption in PND 0 Neonates

We selected genes that are responsible for maintaining the integrity of the BBB, including components such as tight junctions, transporters, and tanycytes. All BBB interaction effects can be seen in Supplementary Table S3. In Table 2, expression of *Claudin-3* was affected in both males and females with greater than a ~2-fold increase in expression in both diets after perinatal OPFR treatment (*p* < 0.05). The opposite effect was observed for *Claudin-5* expression, where decreases in expression were observed in the same groups as *Claudin-3* (*p* < 0.05). Expression of *Dysferlin* was affected by sex in the LFD OPFR group, with an increase in males (*p* < 0.05). *Lrp1* expression was affected by treatment and sex, as well as an interaction between diet and treatment. In females, *Lrp1* expression was higher in HFD oil neonates compared to HFD OPFR neonates (*p* < 0.05), and the female LFD oil group had higher expression than the male LFD oil group (*p* < 0.05). We saw an effect of treatment where perinatal OPFR treatment induced a more than ~4-fold increase in *Lrp2* expression for all sex and diet groups (*p* < 0.05). We also observed an increase in *Lrp2* expression in female HFD OPFR neonates compared to male HFD OFPR (*p* < 0.05).

Treatment effects were also observed in *Occludin* expression, such that perinatal OPFR treatment induced a more than ~4-fold increase in expression for all sex and diet groups (*p* < 0.05). In males, *Occludin* expression was higher in the HFD OPFR group compared to the LFD OPFR group (*p* < 0.05). An interaction of sex, diet, and treatment was found in *Plvap* expression (Table S3). In LFD neonates, there was a more than ~2-fold increase in *Plvap* expression in oil-treated females compared to OPFR-treated females and oil-treated males (*p* < 0.05). In males, *Plvap* expression was ~4-fold lower in the HFD OPFR group compared to the LFD OPFR group (*p* < 0.05). We also saw a treatment effect in HFD males where OPFR-treated neonates expressed *Plvap* ~5-fold lower than oil-treated neonates (*p* < 0.05). In *Vimentin* expression, we observed an effect of treatment and diet, along with an interaction of sex, diet, and treatment (Table S3). In females, *Vimentin* expression was higher in oil-treated neonates compared to OPFR-treated neonates in both diets (*p* < 0.05). Female oil LFD neonates had higher *Vimentin* expression than their male counterpart (*p* < 0.05). In *Zo1* expression, we observed an effect of sex in LFD oil neonates and HFD OPFR neonates with a higher expression in females than males in each respective group (*p* < 0.05).

### 3.4. Hypothalamic Gene Expression in PND 0 and PND 14 Neonates

We also examined genes responsible for energy homeostasis and reproduction in the hypothalamus. All the following gene expression results are included in Table 3 and interaction effects can be found in Supplementary Table S3.

#### 3.4.1. Neuropeptides

In PND 0 neonates, no differences were observed for mRNA levels of *Agrp*, *Cart*, or *Npy*; however there was a sex and diet interaction effect for *Pomc* (Table S3). In PND 0 females, LFD oil *Pomc* expression was higher than HFD oil neonates (*p* < 0.05), and both LFD treatment groups had higher expression than their male counterparts (*p* < 0.05). In PND 14 pups, *Npy* expression was affected by two interaction effects: sex and diet, and treatment and diet. Expression of *Npy* was increased in HFD oil females compared to HFD OPFR females and HFD oil males (*p* < 0.05). Male LFD OPFR pups also had increased *Npy* expression compared to male HFD OPFR pups (*p* < 0.05). In PND 14 pups, *Agrp* and *Cart* levels were increased in HFD oil males compared to HFD OPFR males, while *Pomc* expression was decreased in the same groups (*p* < 0.05). In both males and females, *Pomc* expression was decreased in LFD oil neonates compared to LFD OPFR neonates (*p* < 0.05). In PND 14 pups, expression of *Agrp* was higher in LFD oil males than in HFD oil males (*p* < 0.05). In PND 14 pups, *Cart* expression was higher in LFD oil males than LFD OPFR males (*p* < 0.05). In females, perinatal OPFR treatment decreased *Cart* expression by ~2-fold in HFD females (*p* < 0.05). In *Cart* expression, an effect of sex was also observed in the LFD oil groups (*p* < 0.05).

#### 3.4.2. KNDy Neurons

In PND 0 neonates, we observed an interaction effect of sex, diet, and treatment in *Kiss1* expression (Table S3). Sex differences were noted in all diet and treatment groups, with a more than 5-fold increase in PND 0 females (*p* < 0.05). In PND 0 females, perinatal OPFR treatment decreased Kiss1 expression in both diets (*p* < 0.05). Also, there was a ~2-fold reduction in *Kiss1* expression in HFD oil females compared to LFD oil females. In PND 0 neonates, we observed an interaction effect of sex and treatment in *Tac2* expression (Table S3). Sex differences were noted in all diet and treatment groups, with a more than 2-fold increase in PND 0 females (*p* < 0.05). In PND 0 females, perinatal OPFR treatment decreased *Tac2* expression in both diets (*p* < 0.05). In PND 0 neonates, we observed an interaction effect of sex, diet, and treatment in *Bdnf* and *Foxo1* expression (Table S3). In *Bdnf* expression, sex differences were noted in HFD oil and LFD OPFR groups with an increase in PND 0 females (*p* < 0.05). *Foxo1* expression was increased by perinatal OPFR treatment in female LFD neonates and male HFD neonates (*p* < 0.05). In PND 0 neonates, *Pdyn* expression was affected by OPFR with higher expression in HFD OPFR females compared to HFD oil females.

In PND 14 pups, we observed an interaction effect of diet and treatment in *Bdnf* expression (Table S3). In *Bdnf* and *Foxo1* expression, treatment differences were found in both diet groups with lower expression in OPFR males compared to oil males (*p* < 0.05). In *Bdnf* and *Foxo1* expression, treatment differences were also found in female HFD groups with ~2-fold decrease in OPFR (*p* < 0.05). While there was also a ~2-fold decrease in *Foxo1* expression in PND 14 LFD OPFR females compared to oils, only a sex difference was noted in LFD OPFR females for *Bdnf* expression (*p* < 0.05). In *Kiss1* expression, a sex effect was noted in the LFD OFPR group, and a treatment effect was observed in the HFD males with over ~2-fold reduction in the OPFR group (*p* < 0.05). In PND 14 pups, *Pdyn* expression was affected by OPFR with lower expression in HFD OPFR females compared to HFD oil females. In PND 14 pups, *Tac2* expression was affect by treatment with lower expression in OPFR-treated HFD females and LFD males.

#### 3.4.3. Receptors

In PND 0 neonates, we observed an interaction effect of diet and treatment in *Ghsr* expression (Table S3). Expression of *Ghsr* was increased in HFD OPFR pups compared to LFD OPFR pups in both sexes (*p* < 0.05). A sex effect was also observed in LFD oil groups with higher *Ghsr* expression in females than males (*p* < 0.05). We observed an effect of treatment where perinatal OPFR treatment induced a ~2-fold increase in *Insr* expression for all sex and diet groups (*p* < 0.05). In PND 0 neonates, we noted sex differences with an increase in female expression of Esr1 in the LFD oil group and *Lepr* in the HFD OPFR group (*p* < 0.05).

In PND 14 pups, we observed an interaction effect of sex, diet, and treatment in *Esr1* expression (Table S3). Expression of Esr1 was higher in HFD oil females compared to HFD OPFR and LFD oil female groups (*p* < 0.05). In PND 14 pups, we observed interaction effects of sex and treatment and diet and treatment in *Insr* expression (Table S3). Oil-treated females had increased *Insr* expression compared to males in both diets. In PND 14 pups, two treatment differences were observed in *Insr* expression: HFD oil females had higher expression than HFD OPFR females, and LFD oil males had lower expression than LFD OPFR males. In PND 14 pups, we observed an interaction effect of sex and treatment in *Pparg* expression (Table S3). In *Pparg* expression, HFD oil males had a more than 4-fold increase compared to HFD oil females, in addition to a ~5-fold increase compared to HFD OPFR males. In *Ghsr* and *Lepr* expression, treatment differences were found in HFD females with lower expression in HFD OPFR females (*p* < 0.05). Similarly, HFD OPFR males had lower *Lepr* expression compared to HFD oil males. Expression of *Lepr* was decreased in HFD OPFR pups compared to LFD OPFR pups in both sexes (*p* < 0.05).

### 3.5. Hepatic Gene Expression in PND 0 and PND 14 Neonates

We analyzed the expression of genes responsible for glucose, fatty acid, triglyceride, and xenobiotic metabolism in the liver. All interaction effects can be found in Supplementary Table S4.

#### 3.5.1. Receptors

All HNR gene expression results can be found in Table 4, and all interaction effects can be found in Table S4. In PND 0 females, *Esr1* expression was higher in LFD oil neonates than HFD oil neonates (*p* < 0.05). *Esr1* expression was also higher in LFD OPFR females than HFD OPFR females (*p* < 0.05). In PND 0 pups, we observed an interaction effect of sex and diet on *Insr* expression. *Insr* expression was ~2-fold higher in HFD oil males than HFD OPFR males (*p* < 0.05). In PND 0 neonates, we observed an interaction effect of sex, diet, and treatment on *Lepr* expression. Sex differences were noted in HFD groups, and OPFR treatment increased *Lepr* expression over 3-fold in HFD females (*p* < 0.05). In PND 0 neonates, we observed an interaction effect of sex and diet on *Pparg* expression.

In PND 14 pups, *Esr1* expression was higher in LFD oil males compared to LFD OPFR males, HFD oil males, and LFD oil females (*p* < 0.05). *Lepr* expression in PND 14 pups was affected by treatment and diet, with over 2-fold higher expression in LFD OPFR females compared to LFD oil females and HFD OPFR females.

#### 3.5.2. Enzymes

All metabolic enzyme gene expression results can be found in Table 4, and all interaction effects can be found in Table S4. In *Fasn* expression, OPFR treatment suppressed HFD female expression ~2-fold compared to LFD females (*p* < 0.05). In PND 0 neonates, we observed an interaction effect of sex, diet, and treatment on *G6pc* expression. In PND 0 neonates, we observed an interaction effect of sex and diet on *Pepck* expression. *Pepck* expression was higher in HFD OPFR females compared to HFD OPFR males (*p* < 0.05).

In PND 14 pups, *Dgat2* expression was higher in LFD oil males compared to HFD oil males and LFD oil females (*p* < 0.05). Similarly, in PND 14 pups, *Foxo1* expression was higher in LFD oil males compared to their female counterparts (*p* < 0.05).

#### 3.5.3. Xenobiotic Targets

All xenobiotic gene expression results can be found in Table 5, and all interaction effects can be found in Table S4. In PND 0 neonates, we observed an interaction effect of sex and diet on *Cd36* expression. *Cd36* expression in PND 0 neonates was affected by sex with lower expression in HFD oil females compared to HFD oil males (*p* < 0.05). In PND 0 neonates, we observed an interaction effect of diet and treatment on *Cyp2b10* expression. *Cyp2b10* expression was over 4-fold higher in HFD OPFR females compared to LFD OPFR females and HFD oil females (*p* < 0.05). In addition, *Cyp2b10* expression was ~2-fold higher in HFD OPFR females compared to their male counterparts (*p* < 0.05). In PND 0 neonates, we observed an interaction effect of sex, diet, and treatment on *Cyp3a11* expression. *Cyp3a11* expression was over 2-fold higher in HFD OPFR females compared to LFD OPFR females and HFD oil females (*p* < 0.05). *Cyp3a11* expression was ~3-fold higher in HFD oil males compared to LFD oil males (*p* < 0.05). In PND 0 neonates, *Cyp4a10* expression was affected by OPFR treatment, where HFD OPFR females had a ~5-fold increase in expression compared to HFD oil females (*p* < 0.05). In PND 0 neonates, we observed an interaction effect of sex and diet on *Ostb* expression; however, no pairwise differences were noted. In PND 0 neonates, we observed an interaction effect of diet and treatment on *Shp* expression. *Shp* expression was over 4-fold higher in HFD OPFR females compared to LFD OPFR females, HFD oil females, and HFD OPFR males (*p* < 0.05).

In PND 14 pups, we observed an interaction effect of sex, diet, and treatment on *Cyp4a10* expression. *Cyp4a10* expression in PND 14 pups was affected by diet and sex, with higher expression in LFD oil males compared to HFD oil males (~3-fold) and LFD oil females (~2-fold) (*p* < 0.05). In PND 14 pups, the expression of *Bsep*, *Cyp3a11*, and *Cyp4a10* was decreased in HFD OPFR females compared to LFD OPFR females (*p* < 0.05). *Cyp3a11* expression was lower in HFD oil males compared to LFD oil males (*p* < 0.05). *Cd36* expression in PND 14 pups was affected by sex, with lower expression in HFD oil females compared to HFD oil males (*p* < 0.05). In PND 14 pups, *Ostb* and *Shp* expression was affected by treatment and sex, such that HFD oil females had higher expression than HFD OPFR females (~2-fold) and HFD oil males (*p* < 0.05).

### 3.6. Adult Offspring Behavior Tests—Avoidance

#### 3.6.1. Open Field Test (OFT)

The OFT was performed to assess exploratory and anxiety-like behaviors. A main effect of treatment was noted for locomotion; however, there were no pairwise differences (Table S6, Figure 3A and Figure S1A). We observed treatment and diet differences in adult female offspring for latency to first entry into the 20 cm center (Figure 3B). LFD OPFR females initially took longer to enter the 20 cm center compared to their oil and HFD counterparts (both: *p* < 0.05). We observed treatment and diet differences in adult female offspring in terms of the percentage of time spent in the 10 cm center zone (Figure 3C). LFD oil females spent a higher percentage of time in the center compared to their OPFR (*p* < 0.01) and HFD (*p* < 0.05) counterparts. We observed treatment and sex differences in adult female offspring for perimeter visits (Figure S1B). LFD OPFR females visited the perimeter more than LFD oil females and LFD OPFR males (both *p* < 0.05).

#### 3.6.2. Elevated Plus Maze (EPM)

The EPM was utilized to measure anxiety-like behavior in adult offspring. Locomotion was affected by perinatal OPFR treatment (Table S6, Figure 3D and Figure S1C). Distance and mean speed increased in LFD OPFR males compared to LFD oil males (*p* < 0.01). LFD OPFR males entered (*p* < 0.01; Figure 3F) and traveled (*p* < 0.0001; Figure 3E) more in the closed arms than their oil counterparts. LFD oil females traveled less distance in the closed arms compared to LFD OPFR females (*p* < 0.05; Figure 3E).

#### 3.6.3. Light/Dark Box Emergence Test (LDB)

LDB was used along with OFT and EPM to analyze exploratory and anxiety-like behavior. A main effect of treatment was noted for locomotion; however there were no pairwise differences (Table S6, Figure 3G and Figure S1D). We observed treatment differences in adult female offspring where LFD OPFR females had lower light zone entries (*p* < 0.05; Figure 3H) and exits (*p* < 0.05; Figure 3I) compared to their oil counterparts.

### 3.7. Adult Offspring Behavior Test—Hippocampal-Dependent Memory

The Y-maze test was performed to understand the impact of perinatal OPFR treatment and maternal diet on hippocampal-dependent memory. Locomotion was not affected by sex, diet, or treatment (Figure 4A,B and Table S6). HFD OPFR females spent a smaller percentage of time in the unknown arm compared to their oil-treated counterparts (*p* < 0.05; Figure 4C). HFD oil females spent a greater percentage of time in the unknown arm than their male counterparts (*p* < 0.05; Figure 4C).

### 3.8. Adult Body Composition

Sex differences were observed across all groups for body weight (*p* < 0.05; Figure 5A). For lean mass, in addition to sex differences across all groups, LFD OPFR females had reduced lean mass compared to their oil-treated counterparts (*p* < 0.05; Figure 5B). Sex differences were present in all groups for fat mass, except in HFD OPFR offspring (*p* < 0.05; Figure 5C).

### 3.9. Adult Metabolic Phenotyping

At nighttime, oxygen consumption (Figure 6A) and carbon dioxide production (Figure 6B) decreased in LFD OPFR females compared to LFD oil females (V.O_2_: *p* < 0.01; V.CO_2_: *p* < 0.0001). Nighttime RER, a measure of substrate utilization (fat vs. carbohydrates), was affected by OPFR in sex and diet groups (Figure 6C). OPFR-treated offspring had a lower RER compared to oil-treated offspring (LFD groups: *p* < 0.0001; HFD males: *p* < 0.001), except for HFD OPFR females having a higher RER compared to LFD OPFR females (*p* < 0.05). At nighttime, energy expenditure (EE; Figure 6D) and wheel running counts (Figure 6E) decreased in LFD OPFR females compared to LFD oil females (EE: *p* < 0.01; wheel: *p* < 0.0001).

### 3.10. Adult Glucose Homeostasis

To determine the effects of perinatal OPFR treatment and maternal diet on glucose homeostasis, we conducted glucose and insulin tolerance tests on all mice. No differences were observed with the glucose tolerance test (GTT), other than HFD OPFR females having a lower AUC compared to their male counterparts (*p* < 0.001; Figure 7A–C). Insulin tolerance in males was affected by treatment and diet (Figure 7D,F). In LFD oil males, insulin-induced glucose clearance levels were higher 15 min postinjection compared with LFD OPFR males (*p* < 0.05; Figure 7D). In HFD oil males, insulin-induced glucose clearance levels were higher 60 and 90 min postinjection compared with HFD OPFR males (*p* < 0.05; Figure 7D). In HFD OPFR males, insulin-induced glucose clearance levels were higher 120 min postinjection compared with LFD OPFR males (*p* < 0.05; Figure 7D). HFD OPFR males had a lower AUC than LFD OPFR males (*p* < 0.05; Figure 7F). In ITT AUC, sex differences were noted in all groups, except for HFD oil (*p* < 0.05; Figure 7F).

## 4. Discussion

Flame retardants remain widely used, with recent studies reporting exposure to both conventional and emerging OPFRs in chorionic villus samples, children, and the environment [5,46,47]. In parallel, preconception obesity rates have risen significantly [22,23,24]. Both environmental contaminants and lifestyle factors can profoundly influence the maternal–fetal environment, potentially resulting in both short- and long-term consequences for offspring health [48]. Investigating the combined effects of maternal diet and perinatal OPFR exposure on offspring development is essential, as evaluating these factors separately does not reflect the complexity of real-world exposures. Increasing evidence from our group and others demonstrates that maternal diet alone can significantly disrupt fetal and neonatal programming, ultimately affecting offspring health outcomes [25,30,38,39,42,43]. We have observed age- and sex-dependent effects in offspring, from PND 0 through adulthood, resulting from impacts on the maternal–fetal environment.

Maternal diet impacted AGD, a measurement commonly used to assess endocrine disruption during genital development [49]. Maternal HFD increased AGD in oil-treated male and female offspring, potentially due to reduced maternal and placental estradiol synthesis, which may shift the intrauterine hormonal balance toward increased androgen exposure [50,51]. Interestingly, we observed a decrease in AGD among HFD-exposed males treated with OPFRs, suggesting that perinatal OPFR exposure may restore intrauterine hormonal balance. Our previous work also demonstrated a treatment effect in male offspring from dams fed a standard chow diet [13]. Although it is important to note that body weights were not taken of the PND 7 mice, the effects seen may be due to maternal HFD or OPFR exposure on pup size.

In utero exposure to OPFRs led to widespread alterations in BBB gene expression at PND 0. Disruption of key BBB components, such as transporters, tanycytic processes, and vascular fenestrations, can increase permeability to the brain [30,52,53]. Perinatal OPFR treatment increased expression of tight junctions *Claudin-3* and *Occludin* across both diet and sex groups, but decreased *Claudin-5*, *Vimentin* (tanycyte marker), and *Plvap* (vascular endothelial cell marker). Leptin transporters *Lrp1* and *Lrp2* were both increased after perinatal OPFR treatment across all groups, with higher *Lrp2* expression in HFD OPFR females compared to males. Studies have shown that OPFRs can cross the BBB in both zebrafish and humans, suggesting that fetal BBB formation is compromised [54,55]. Within OPFR-treated males, maternal HFD further increased *Occludin* expression and decreased *Plvap* expression compared to LFD neonates, consistent with previous findings that maternal obesity disrupts BBB integrity [30].

Increased BBB permeability in the mediobasal hypothalamic (MBH) region may enhance OPFR deposition and alter the development of neural circuits regulating energy balance. For neuropeptide expression, most differences appeared by PND 14, likely due to prolonged OPFR exposure via lactation. At PND 14, expression of *Agrp*, *Cart*, and *Npy* was decreased in HFD OPFR-treated groups compared to HFD oil-treated controls, in a sex-dependent manner. Previously, we observed decreased *Npy* expression only in OPFR-treated males [13]; here, we also noted a decrease in HFD OPFR-treated females. Moreover, *Npy* expression was lower in HFD OPFR-treated males than in LFD OPFR-treated males, suggesting that maternal diet and OPFR exposure jointly disrupt orexigenic pathways. Sex-dependent differences may reflect distinct neuropeptide expression patterns during the transition to chow feeding at PND 14. *Pomc* expression was elevated in OPFR-treated groups among LFD males and females, as well as HFD males. Since POMC promotes anorexigenic signaling, while NPY and AgRP promote orexigenic pathways, these data further demonstrate that maternal diet and OPFR exposure disrupt the regulation of energy homeostasis, likely due to increased BBB permeability.

For genes associated with KNDy neurons, we noted many treatment- and sex-dependent differences in *Kiss1* and *Tac2* expression at PND 0, consistent with their roles in reproduction and sexual maturation [13]. Differences in *Bdnf* expression emerged more prominently at PND 14. Maternal obesity is known to disrupt hippocampal *Bdnf*, essential for neurogenesis and synaptic plasticity [56]. At PND 14, *Bdnf* expression was decreased in HFD OPFR-treated males and females. while LFD oil-treated males showed higher *Bdnf* expression than LFD OPFR-treated males and LFD oil-treated females. Maternal HFD appeared to abolish these sex differences, underscoring the influence of diet on neurodevelopment.

The FOXO transcription factors are known to respond to environmental stimuli, such as insulin, nutrient levels, and oxidative stress, and promote cell survival and neurogenesis [57]. At PND 0, OPFR treatment increased *Foxo1* expression in LFD females and HFD males; however, by PND 14, *Foxo1* expression was decreased across all groups after OPFR treatment. This decrease is consistent with studies showing that OPFR exposure, such as TPP, inhibits FOXO signaling, potentially promoting apoptosis and disrupting energy homeostasis [58].

Maternal obesity and OPFR exposure also disrupted hormone receptor expression. At PND 0, OPFR treatment increased *Insr* expression across all groups, and *Ghsr* expression was elevated in HFD OPFR-treated compared to LFD OPFR-treated mice. By PND 14, most differences were resolved except for *Lepr* expression, which was reduced in HFD OPFR-treated males and females compared to their oil-treated counterparts. Given the crucial role of the postnatal leptin surge in hypothalamic development, our findings suggest that maternal diet and OPFR exposure impair leptin signaling by elevating leptin levels and downregulating receptor expression [59,60,61]. These disruptions are further supported by increased *Pomc* expression at PND 14, as leptin excites POMC neurons via activation of TRPC channels [62]. Impairment of leptin signaling is also supported through increased expression of leptin transporters in the BBB (*Lrp1* and *Lrp2*), suggesting enhanced BBB disruption and energy homeostasis in the context of maternal obesity [13].

At PND 14, *Pparg* expression was higher in HFD oil-treated males compared to HFD oil-treated females and HFD OPFR-treated males. Flame retardants are known to disrupt PPARγ signaling, which regulates lipid metabolism and inflammation in various tissues, such as the liver and brain [18]. Studies have cited increases in PPARγ activity after exposure to flame retardants, which led to increased adiposity, a known characteristic of environmental obesogens [17,18,21,63]. Mechanistically, flame retardants alter PPARγ activity through epigenetic modifications and direct receptor interactions [17,19,20,21]. Given that fatty acids are potent PPARγ ligands, the observed decrease in *Pparg* expression in HFD OPFR males may result from ligand competition of maternal dietary fats [64]. Altered *Pparg* expression may therefore lead to reduced expression of downstream target genes, ultimately disrupting lipid homeostasis and increasing oxidative stress [65]. Notably, we observed no significant changes in hepatic *Pparg* expression, indicating a tissue-specific effect in our two-hit model. More mechanistic studies need to be completed to understand the complex role of PPARγ ligand competition on lipid metabolism in regard to OPFRs and dietary lipids.

We have previously shown that maternal OPFR exposure alone disrupts hepatic genes involved in glucose, fatty acid, and xenobiotic metabolism [13]. For receptors, sex-specific changes in *Lepr* expression were notable; at PND 0, HFD OPFR-treated females exhibited a two-fold increase compared to LFD OPFR-treated and HFD oil-treated neonates, whereas by PND 14, *Lepr* expression was three-fold lower in HFD OPFR-treated females compared to LFD OPFR-treated females. These shifts mirror hypothalamic *Lepr* alterations and support the hypothesis that maternal obesity exacerbates OPFR-induced programming of energy homeostasis.

Changes in liver xenobiotic targets were more pronounced. CAR, PXR, and PPARα are all NHRs in the liver that regulate downstream targets, including *Cyp2b10*, *Cyp3a11*, and *Cyp4a10*, respectively. In PND 0 females, expression of these targets was elevated in the HFD OPFR group compared to the HFD oil group (over 3-fold) and LFD OPFR group (over 2-fold). However, by PND 14, *Bsep*, *Cyp3a11*, and *Cyp4a10* expression was reduced in HFD OPFR females relative to LFD OPFR females. Previously, maternal OPFR exposure alone modestly increased *Cyp2b10* and *Cyp4a10* expression [13], indicating that maternal obesity sensitizes offspring hepatic gene regulation to OPFRs in a sex-specific manner. Additionally, *Ostb* and *Shp* expression was lower in HFD OPFR females compared to HFD oil females, indicating disruption of bile acid metabolism and secretion.

Maternal HFD has previously been shown to alter the expression of bile acid regulators in offspring, which can ultimately affect liver physiology and contribute to steatosis [66]. Notably, BSEP is a known target of the organophosphate flame retardant tri-ortho-cresyl phosphate (TOCP), which was found to be downregulated in HepaRG cells and primary human hepatocytes, further supporting OPFR-associated hepatotoxicity [67]. Others have also reported modulation of gut microbiome composition following treatment with TPP, which correlated with alterations in bile acid pools [68] and reduced expression of bile acid markers such as *Shp* [69]. Bile acids are ligands for FXR, a NHR that regulates bile acid metabolism by controlling expression of targets including *Bsep*, *Ostb*, and *Shp* [70]. The observed alterations in these markers suggest that the bile acid metabolism pathway is disrupted during development, particularly under conditions of maternal HFD and OPFR exposure.

In adulthood, maternal LFD OPFR-treated offspring displayed increased anxiogenic phenotypes compared to their oil counterparts, with locomotor differences contributing to these phenotypes. Across all avoidance tests, a main effect of treatment was noted for distance traveled and mean speed. Our previous work using mice and that of other groups using rats demonstrated that maternal OPFR exposure influences locomotor and avoidance behaviors in males [71,72]. Notably, maternal OPFR treatment alone produced locomotor differences, leading to an anxiolytic phenotype in males on the EPM, but an anxiogenic phenotype on the OFT. The present findings align with these prior observations, underscoring the influence of motor activity on behavioral parameters measured, such as entry to latency in the OFT, distance traveled in the closed arm of the EPM, and light zone entries in the LDB. Collectively, our results reveal sex-specific anxiogenic phenotypes in LFD OPFR-treated offspring that are modulated by treatment-induced locomotor effects.

We assessed hippocampal-dependent memory using the Y-maze test. No differences in locomotion were noted. However, HFD OPFR-treated females spent less time in the unknown arm compared to their oil counterparts. Cognitive deficits have been reported in offspring maternally treated with HFD or OPFRs, which are driven by mechanisms such as disrupted neurogenesis and synaptic dysfunction [73,74,75]. Interestingly, studies have found associations between impairments in recognition memory and both peripheral and central leptin resistance, with one study even demonstrating that leptin signaling directly mediates hippocampal spine formation, a process linked to neurogenesis and synaptic plasticity [76,77,78,79]. We observed alterations in *Lepr* expression from PND 0 through PND 14 in the liver of female neonates, and at PND 14 in the hypothalamus of both female and male neonates. Based on these observations, we hypothesize that disrupted leptin signaling underlies the cognitive impairments observed in HFD OPFR-treated females. However, more direct measurements of leptin signaling would be needed to fully elucidate this mechanism.

Body composition in adulthood was largely unaffected, except for decreased lean mass in LFD OPFR-treated females. One possible reason for the lack of significant treatment and diet differences in body composition in adulthood could be that long-term feeding on a normal chow diet abrogated any negative effects from maternal diet or treatment. The decrease in lean mass was correlated with reduced nighttime oxygen consumption, carbon dioxide production, respiratory exchange ratio, energy expenditure, and wheel-running counts. We hypothesize that the loss in lean mass is due to perturbations in metabolism and energy usage at nighttime, when mice are most active. We previously observed this same trend in maternally dosed OPFR female mice that were fed LFD in adulthood [35]. No significant differences were observed in GTTs, but ITTs revealed improved glucose clearance in HFD OPFR-treated males compared to LFD OPFR-treated males. This same effect was also observed in our previous study in maternally dosed OPFR male mice that were fed an HFD in adulthood [35]. Perhaps we would have seen more differences in glucose response using more sensitive techniques, such as the hyperinsulinemic euglycemic clamp. These consistent outcomes highlight the long-term metabolic impacts of perinatal OPFR exposure.

## 5. Conclusions

Our findings align with the developmental origins of health and disease (DOHaD) hypothesis, which posits that early life exposures can program long-term health outcomes. We identified the early postnatal period as a critical window of vulnerability in mice, particularly in response to maternal diet, during which females showed more frequent and sometimes stronger alterations than males. Female offspring demonstrated greater sensitivity to maternal diet and OPFR exposure, especially in hypothalamic and hepatic gene expression, consistent with observations from our previous publication [13]. A disrupted BBB can have deleterious effects on the development of the offspring, which would increase their susceptibility to blood-borne molecules. Our data further support the liver as a sensitive site for metabolic programming influenced by the maternal environment, which has implications for bile homeostasis and drug metabolism, thus contributing to long-term metabolic diseases. These findings suggest that early disruptions in BBB formation and energy homeostasis may underlie the persistent adult phenotypes observed in behavioral, metabolic, and cognitive parameters.

We found that LFD OPFR-treated females exhibited increased anxiogenic behaviors and poorer metabolic outcomes, while HFD OPFR-treated males showed improved insulin-induced glucose clearance, suggesting that maternal HFD may offer some protective effects against the impacts of maternal OPFR treatment. Although we anticipated greater differences in the HFD OPFR-treated groups in adulthood based on our neonatal findings, it is possible that the normal chow diet they received mitigated effects that might have emerged had the mice been maintained on HFD instead, especially since our previous study has shown that maternal HFD potentiated the effects of adult HFD [43]. It is also possible that the maternal diet provided protective long-term effects on the offspring. While we saw additive effects of OPFR and HFD exposure during the neonatal period, we saw that these effects were mostly abolished at adulthood, with only cognition being affected by these two factors. Our findings underscore the importance of minimizing gestational exposures to environmental toxicants and maintaining a healthy maternal diet to reduce long-term disease risk in offspring.

## Figures and Tables

**Table 1:** Dam and litter size per group. * = one dam was used twice because only one sex was birthed in the first litter.

	LFD		HFD	
	Oil	OPFR	Oil	OPFR
**Dams**	10 *	10	10	14
**Litters**	11	10	10	14

**Table 2:** Hypothalamic gene expression in PND 0 neonates—Blood–Brain Barrier (BBB). Relative gene expression in PND 0 female and male pups from dams on an LFD or HFD and orally dosed with vehicle or OPFR mixture. Data are presented as mean ± SEM and analyzed by litter (n = 8–12/group) (**^b^** = *p* < 0.05 for LFD oil and LFD OPFR; **^c^** = *p* < 0.05 for HFD oil and HFD OPFR; **^d^** = *p* < 0.05 for LFD OPFR and HFD OPFR; **^e^** = sex difference within the diet and treatment).

			Females		Males	
Gene	Role	Maternal Diet	Oil	OPFR	Oil	OPFR
***Claudin-3***	Tight junction	LFD	0.4 ± 0.1 **^b^**	1.9 ± 0.5	0.7 ± 0.1 **^b^**	2.1 ± 0.3
		HFD	0.9 ± 0.2 **^c^**	2.3 ± 0.4	0.8 ± 0.1 **^c^**	1.8 ± 0.4
***Claudin-5***	Tight junction	LFD	21.2 ± 1.0 **^b,e^**	12.5 ± 0.6	16.8 ± 1.8 **^b^**	11.7 ± 1.8
		HFD	20.9 ± 0.6 **^c^**	11.4 ± 1.7	19.7 ± 1.3 **^c^**	10.3 ± 1.1
***Dysferlin***	Endothelial marker	LFD	0.9 ± 0.2	0.9 ± 0.1 **^e^**	0.8 ± 0.1	1.3 ± 0.2
		HFD	0.5 ± 0.2	1.0 ± 0.1	0.8 ± 0.1	1.0 ± 0.1
***Lrp1***	Leptin transporter	LFD	0.9 ± 0.0 **^e^**	0.7 ± 0.1	0.6 ± 0.1	0.6 ± 0.1
		HFD	1.1 ± 0.2 **^c^**	0.7 ± 0.1	0.9 ± 0.1	0.6 ± 0.1
***Lrp2***	Leptin transporter	LFD	0.4 ± 0.1 **^b^**	1.7 ± 0.4	0.4 ± 0.0 **^b^**	2.0 ± 0.5
		HFD	0.5 ± 0.1 **^c^**	2.6 ± 0.4 **^e^**	0.4 ± 0.1 **^c^**	1.6 ± 0.4
***Occludin***	Tight junction	LFD	0.5 ± 0.1 **^b^**	2.9 ± 0.5	0.5 ± 0.0 **^b^**	2.0 ± 0.3 **^d^**
		HFD	0.8 ± 0.1 **^c^**	3.2 ± 0.4	0.5 ± 0.1 **^c^**	3.3 ± 0.5
***Plvap***	Endothelial marker	LFD	0.7 ± 0.1 **^b,e^**	0.3 ± 0.1	0.3 ± 0.1	0.4 ± 0.1 **^d^**
		HFD	0.6 ± 0.1	0.3 ± 0.1	0.5 ± 0.1 **^c^**	0.1 ± 0.0
***Vimentin***	Tanycytic processes	LFD	1.6 ± 0.1 **^b,e^**	1.0 ± 0.1	0.8 ± 0.1	1.1 ± 0.1
		HFD	1.3 ± 0.1 **^c^**	0.9 ± 0.1	1.1 ± 0.1	0.8 ± 0.1
***Zo1***	Tight junction	LFD	1.2 ± 0.1 **^e^**	0.8 ± 0.1	0.6 ± 0.1	0.8 ± 0.1
		HFD	1.2 ± 0.1	1.1 ± 0.2 **^e^**	0.9 ± 0.1	0.7 ± 0.1

**Table 3:** Hypothalamic gene expression in PND 0 and PND 14 neonates—receptors and KNDy neurons. Relative gene expression in PND 0 and PND 14 female and male pups from dams on an LFD or HFD and orally dosed with vehicle or OPFR mixture. Data are presented as mean ± SEM and analyzed by litter (n = 8–12/group) (**^a^** = *p* < 0.05 for LFD oil and HFD oil; **^b^** = *p* < 0.05 for LFD oil and LFD OPFR; **^c^** = *p* < 0.05 for HFD oil and HFD OPFR; **^d^** = *p* < 0.05 for LFD OPFR and HFD OPFR; **^e^** = sex difference within the diet and treatment).

			PND 0				PND 14			
			Females		Males		Females		Males	
Gene	Role	Maternal Diet	Oil	OPFR	Oil	OPFR	Oil	OPFR	Oil	OPFR
***Agrp***	Orexigenic neuropeptide	LFD	1.2 ± 0.2	0.8 ± 0.1	1.0 ± 0.2	0.5 ± 0.1	1.1 ± 0.2	0.7 ± 0.1	0.9 ± 0.2 **^a^**	0.8 ± 0.1
		HFD	0.8 ± 0.2	0.9 ± 0.2	0.9 ± 0.2	0.8 ± 0.1	1.2 ± 0.2	0.6 ± 0.1	1.6 ± 0.3 **^c^**	0.7 ± 0.1
***Bdnf***	Synaptic plasticity	LFD	1.1 ± 0.1	1.0 ± 0.0 **^e^**	1.0 ± 0.0	0.8 ± 0.1	0.8 ± 0.1 **^e^**	0.7 ± 0.0	1.1 ± 0.1 **^b^**	0.7 ± 0.0
		HFD	1.3 ± 0.1 **^e^**	1.1 ± 0.1	0.8 ± 0.1	1.0 ± 0.1	1.0 ± 0.1 **^c^**	0.5 ± 0.0	1.1 ± 0.1 **^c^**	0.6 ± 0.0
***Cart***	Anorexigenic neuropeptide	LFD	1.0 ± 0.0	0.9 ± 0.0	0.8 ± 0.1	1.0 ± 0.1	0.7 ± 0.1 **^e^**	0.6 ± 0.1	1.1 ± 0.1 **^b^**	0.6 ± 0.1
		HFD	0.9 ± 0.1	1.1 ± 0.1	0.7 ± 0.1	1.0 ± 0.1	1.0 ± 0.2 **^c^**	0.5 ± 0.0	1.1 ± 0.1 **^c^**	0.7 ± 0.1
***Esr1***	Energy homeostasis	LFD	1.9 ± 0.2 **^e^**	1.5 ± 0.0	1.2 ± 0.2	1.1 ± 0.2	1.3 ± 0.1 **^a^**	1.1 ± 0.1	1.1 ± 0.1	1.0 ± 0.1
		HFD	1.4 ± 0.3	1.5 ± 0.2	1.1 ± 0.1	1.1 ± 0.1	2.2 ± 0.4 **^c,e^**	0.9 ± 0.1	0.9 ± 0.1	1.0 ± 0.1
***Foxo1***	Energy homeostasis	LFD	1.0 ± 0.1 **^b^**	1.5 ± 0.2	1.3 ± 0.1	1.2 ± 0.1	1.3 ± 0.2 **^b^**	0.6 ± 0.0	1.1 ± 0.2 **^b^**	0.6 ± 0.0
		HFD	1.3 ± 0.2	1.4 ± 0.1	1.1 ± 0.1 **^c^**	1.4 ± 0.1	1.4 ± 0.1 **^c^**	0.5 ± 0.0	1.2 ± 0.1 **^c^**	0.7 ± 0.1
***Ghsr***	Ghrelin receptor	LFD	0.8 ± 0.1 **^e^**	0.7 ± 0.1 **^d^**	0.5 ± 0.1	0.5 ± 0.1 **^d^**	0.9 ± 0.1	0.8 ± 0.1	1.0 ± 0.1	1.0 ± 0.1
		HFD	0.8 ± 0.2	1.1 ± 0.1	0.7 ± 0.1	0.9 ± 0.1	1.0 ± 0.1 **^c^**	0.7 ± 0.1	0.9 ± 0.1	0.8 ± 0.1
***Insr***	Insulin receptor	LFD	1.1 ± 0.1 **^b^**	1.8 ± 0.2	1.0 ± 0.1 **^b^**	1.7 ± 0.1	0.9 ± 0.1 **^e^**	0.9 ± 0.0	0.6 ± 0.0 **^b^**	0.8 ± 0.1
		HFD	1.1 ± 0.0 **^c^**	1.9 ± 0.1	0.8 ± 0.1 **^c^**	1.6 ± 0.1	1.0 ± 0.1 **^c,e^**	0.8 ± 0.1	0.7 ± 0.1	0.9 ± 0.0
***Kiss1***	Reproduction	LFD	10 ± 0.7 **^a,b,e^**	3.1 ± 0.5 **^e^**	1.3 ± 0.2	0.6 ± 0.1	1.6 ± 0.2	1.5 ± 0.1 **^e^**	1.5 ± 0.3	0.9 ± 0.1
		HFD	4.7 ± 0.8 **^c,e^**	2.5 ± 0.6 **^e^**	1.1 ± 0.2	0.4 ± 0.1	1.6 ± 0.3	0.9 ± 0.2	1.4 ± 0.2 **^c^**	0.6 ± 0.1
***Lepr***	Leptin receptor	LFD	1.4 ± 0.2	1.4 ± 0.3	1.1 ± 0.2	1.2 ± 0.2	0.8 ± 0.1	0.7 ± 0.1 **^d^**	1.0 ± 0.1	0.8 ± 0.1 **^d^**
		HFD	1.1 ± 0.2	1.6 ± 0.1 **^e^**	0.9 ± 0.1	0.9 ± 0.1	0.8 ± 0.1 **^c^**	0.5 ± 0.1	0.9 ± 0.1 **^c^**	0.6 ± 0.0
***Npy***	Orexigenic neuropeptide	LFD	1.5 ± 0.1	1.6 ± 0.1	1.1 ± 0.2	1.3 ± 0.2	1.1 ± 0.1	1.0 ± 0.1	0.9 ± 0.1	1.1 ± 0.1 **^d^**
		HFD	1.6 ± 0.5	2.2 ± 0.4	1.1 ± 0.1	2.0 ± 0.3	1.2 ± 0.1 **^c,e^**	0.7 ± 0.1	0.8 ± 0.1	0.8 ± 0.0
***Pdyn***	Energy homeostasis	LFD	1.0 ± 0.1	1.1 ± 0.1	0.8 ± 0.1	0.9 ± 0.1	0.9 ± 0.1	0.8 ± 0.1	0.9 ± 0.1	1.0 ± 0.0
		HFD	0.9 ± 0.1 **^c^**	1.1 ± 0.1	0.8 ± 0.1	1.1 ± 0.1	1.1 ± 0.1 **^c^**	0.8 ± 0.1	1.0 ± 0.1	0.9 ± 0.1
***Pomc***	Anorexigenic neuropeptide	LFD	1.0 ± 0.1 **^a,e^**	0.9 ± 0.1 **^e^**	0.6 ± 0.1	0.6 ± 0.1	1.7 ± 0.2 **^b^**	3.2 ± 0.2	1.9 ± 0.4 **^b^**	3.3 ± 0.2
		HFD	0.6 ± 0.1	0.7 ± 0.2	0.6 ± 0.1	0.7 ± 0.1	2.0 ± 0.4	2.5 ± 0.3	2.2 ± 0.4 **^c^**	3.4 ± 0.4
***Pparg***	Energy homeostasis	LFD	0.4 ± 0.1	0.7 ± 0.1	0.4 ± 0.1	0.6 ± 0.1	0.9 ± 0.4	0.5 ± 0.0	2.1 ± 0.7	0.6 ± 0.1
		HFD	0.5 ± 0.1	0.6 ± 0.1	0.6 ± 0.2	0.4 ± 0.1	0.8 ± 0.3 **^e^**	0.4 ± 0.1	3.4 ± 1.4 **^c^**	0.7 ± 0.1
***Tac2***	Sexual maturation	LFD	5.5 ± 0.4 **^b,e^**	2.5 ± 0.2 **^e^**	1.5 ± 0.2	0.9 ± 0.1	1.4 ± 0.1	1.2 ± 0.1	1.4 ± 0.2 **^b^**	0.8 ± 0.0
		HFD	4.5 ± 0.9 **^c,e^**	2.7 ± 0.4 **^e^**	1.2 ± 0.2	0.9 ± 0.1	1.6 ± 0.3 **^c^**	0.9 ± 0.1	1.2 ± 0.1	0.9 ± 0.1

**Table 4:** Hepatic gene expression in PND 0 and PND 14 neonates—receptors and enzymes. Relative gene expression in PND 0 and PND 14 female and male pups from dams on an LFD or HFD and orally dosed with vehicle or OPFR mixture. Data are presented as mean ± SEM and analyzed by litter (n = 8–12/group) (**^a^** = *p* < 0.05 for LFD oil and HFD oil; **^b^** = *p* < 0.05 for LFD oil and LFD OPFR; **^c^** = *p* < 0.05 for HFD oil and HFD OPFR; **^d^** = *p* < 0.05 for LFD OPFR and HFD OPFR; **^e^** = sex difference within the diet and treatment).

			PND 0				PND 14			
			Females		Males		Females		Males	
Gene	Role	Maternal Diet	Oil	OPFR	Oil	OPFR	Oil	OPFR	Oil	OPFR
***Dgat2***	Triglyceride synthesis	LFD	1.0 ± 0.1	0.8 ± 0.1	1.0 ± 0.1	0.8 ± 0.0	1.1 ± 0.2 **^e^**	1.3 ± 0.1	1.6 ± 0.2 **^a^**	1.5 ± 0.1
		HFD	0.8 ± 0.1	0.9 ± 0.1	1.0 ± 0.2	0.7 ± 0.1	1.2 ± 0.1	1.1 ± 0.1	1.1 ± 0.1	1.3 ± 0.1
***Esr1***	Lipid and	LFD	1.2 ± 0.1 **^a^**	1.4 ± 0.2 **^d^**	1.3 ± 0.1	1.2 ± 0.2	1.0 ± 0.1 **^e^**	1.2 ± 0.1	1.5 ± 0.3 **^a,b^**	1.1 ± 0.0
	glucose metabolism	HFD	0.7 ± 0.1	0.9 ± 0.1	0.9 ± 0.2	0.8 ± 0.1	1.1 ± 0.1	1.3 ± 0.1	1.1 ± 0.1	1.3 ± 0.1
***Fasn***	Lipogenesis	LFD	1.1 ± 0.1	1.2 ± 0.2 **^d^**	0.8 ± 0.1	0.9 ± 0.1	1.0 ± 0.1	0.7 ± 0.1	1.3 ± 0.3	0.9 ± 0.2
		HFD	0.7 ± 0.1	0.6 ± 0.1	0.6 ± 0.1	1.0 ± 0.3	1.1 ± 0.1	0.9 ± 0.2	0.9 ± 0.1	1.0 ± 0.1
***Foxo1***	Lipogenesis and	LFD	1.4 ± 0.4	1.3 ± 0.1	1.1 ± 0.2	1.1 ± 0.2	1.1 ± 0.2 **^e^**	1.6 ± 0.4	2.0 ± 0.5	1.2 ± 0.1
	glucose metabolism	HFD	1.3 ± 0.7	1.5 ± 0.2	0.8 ± 0.3	1.0 ± 0.2	1.4 ± 0.1	1.5 ± 0.3	1.4 ± 0.1	1.6 ± 0.4
***G6pc***	Glucose metabolism	LFD	1.2 ± 0.2	0.9 ± 0.1	0.8 ± 0.1	0.8 ± 0.0	1.1 ± 0.2	1.2 ± 0.1	1.3 ± 0.2	1.1 ± 0.2
		HFD	0.9 ± 0.2	1.4 ± 0.2	1.4 ± 0.3	0.9 ± 0.2	1.0 ± 0.1	1.0 ± 0.1	0.9 ± 0.1	1.0 ± 0.1
***Insr***	Insulin receptor	LFD	1.0 ± 0.1	1.0 ± 0.1	1.2 ± 0.2	0.8 ± 0.1	1.0 ± 0.1	1.0 ± 0.1	1.1 ± 0.2	1.0 ± 0.1
		HFD	1.0 ± 0.2	1.1 ± 0.1	1.4 ± 0.2 **^c^**	0.8 ± 0.1	1.0 ± 0.1	1.0 ± 0.1	0.9 ± 0.0	1.0 ± 0.1
***Lepr***	Leptin receptor	LFD	0.8 ± 0.1	0.8 ± 0.1 **^d^**	1.0 ± 0.1	0.7 ± 0.0	1.2 ± 0.3 **^b^**	2.8 ± 0.4 **^d^**	2.1 ± 0.5	2.5 ± 0.4
		HFD	0.5 ± 0.1 **^c,e^**	1.8 ± 0.2 **^e^**	1.0 ± 0.2	1.0 ± 0.1	1.6 ± 0.6	1.0 ± 0.3	1.0 ± 0.3	1.4 ± 0.3
***Pepck***	Gluconeogenesis	LFD	1.1 ± 0.2	1.0 ± 0.1	1.1 ± 0.2	0.7 ± 0.0	1.1 ± 0.2	1.0 ± 0.2	1.1 ± 0.2	0.9 ± 0.2
		HFD	0.8 ± 0.1	1.2 ± 0.2 **^e^**	0.9 ± 0.1	0.8 ± 0.2	1.2 ± 0.1	0.9 ± 0.1	0.8 ± 0.1	0.9 ± 0.1
***Ppara***	Fatty acid oxidation	LFD	1.0 ± 0.2	1.1 ± 0.2	1.4 ± 0.2	0.8 ± 0.1	1.0 ± 0.1	1.1 ± 0.1	1.2 ± 0.3	1.0 ± 0.0
		HFD	1.5 ± 0.4	1.2 ± 0.1	1.5 ± 0.2	0.8 ± 0.1	1.2 ± 0.1	1.0 ± 0.1	1.1 ± 0.1	1.3 ± 0.0
***Pparg***	Lipid metabolism	LFD	1.0 ± 0.1	1.2 ± 0.2	1.2 ± 0.1	0.9 ± 0.1	1.0 ± 0.1	1.0 ± 0.1	1.1 ± 0.1	1.2 ± 0.1
		HFD	0.8 ± 0.1	1.1 ± 0.1	1.0 ± 0.2	0.7 ± 0.1	0.9 ± 0.1	0.7 ± 0.1	0.9 ± 0.1	1.0 ± 0.2

**Table 5:** Hepatic gene expression in PND 0 and PND 14 neonates—xenobiotic targets. Relative gene expression in PND 0 and PND 14 female and male pups from dams on an LFD or HFD and orally dosed with vehicle or OPFR mixture. Data are presented as mean ± SEM and analyzed by litter (n = 8–12/group) (**^a^** = *p* < 0.05 for LFD oil and HFD oil; **^c^** = *p* < 0.05 for HFD oil and HFD OPFR; **^d^** = *p* < 0.05 for LFD OPFR and HFD OPFR; **^e^** = sex difference within the diet and treatment).

			PND 0				PND 14			
			Females		Males		Females		Males	
Gene	Role	Maternal Diet	Oil	OPFR	Oil	OPFR	Oil	OPFR	Oil	OPFR
***Bsep***	Bile salt export pump	**LFD**	1.1 ± 0.2	0.8 ± 0.1	1.0 ± 0.1	1.1 ± 0.0	1.0 ± 0.1	0.9 ± 0.1 **^d^**	0.9 ± 0.1	0.9 ± 0.1
		**HFD**	1.0 ± 0.1	0.6 ± 0.1	1.2 ± 0.2	0.8 ± 0.2	0.9 ± 0.1	0.7 ± 0.1	0.8 ± 0.1	0.9 ± 0.0
***Cd36***	Lipid metabolism	**LFD**	0.8 ± 0.1	0.9 ± 0.1	1.1 ± 0.2	0.9 ± 0.1	1.1 ± 0.2 **^e^**	1.3 ± 0.3	1.6 ± 0.2	1.4 ± 0.2
		**HFD**	0.7 ± 0.1 **^e^**	1.0 ± 0.1	1.1 ± 0.1	0.8 ± 0.2	1.1 ± 0.1	0.9 ± 0.1	1.1 ± 0.1	1.3 ± 0.3
***Cyp2b10***	Target of Constitutive	**LFD**	0.9 ± 0.3	1.2 ± 0.2 **^d^**	1.0 ± 0.2	1.0 ± 0.2	1.2 ± 0.3	1.3 ± 0.2	0.8 ± 0.2	1.6 ± 0.5
	Androstane Receptor	**HFD**	0.7 ± 0.2 **^c^**	4.9 ± 1.4 **^e^**	1.9 ± 0.5	2.5 ± 1.3	1.3 ± 0.2	1.0 ± 0.2	1.7 ± 0.5	1.6 ± 0.3
***Cyp3a11***	Target of	**LFD**	1.1 ± 0.2	1.2 ± 0.2 **^d^**	0.6 ± 0.0 **^a^**	0.9 ± 0.1	1.0 ± 0.1	1.4 ± 0.2 **^d^**	1.4 ± 0.3 **^a^**	1.2 ± 0.1
	Pregnane X Receptor	**HFD**	0.8 ± 0.1 **^c,e^**	2.3 ± 0.5	1.7 ± 0.5	1.1 ± 0.4	1.0 ± 0.0	0.8 ± 0.1	1.0 ± 0.1	1.0 ± 0.1
***Cyp4a10***	Target of PPARα	**LFD**	1.3 ± 0.4	1.2 ± 0.3	0.7 ± 0.2	2.3 ± 0.6	1.1 ± 0.1 **^e^**	1.8 ± 0.3 **^d^**	2.1 ± 0.7 **^a^**	1.2 ± 0.2
		**HFD**	0.6 ± 0.1 **^c^**	3.0 ± 0.8	0.6 ± 0.1	1.9 ± 0.7	0.8 ± 0.2	0.6 ± 0.1	0.6 ± 0.1	1.1 ± 0.3
***Cyp7a1***	Target of	**LFD**	1.4 ± 0.4	1.5 ± 0.3	1.6 ± 0.3	1.2 ± 0.2	1.0 ± 0.1	0.9 ± 0.2	0.9 ± 0.0	1.2 ± 0.4
	Farnesoid X Receptor	**HFD**	1.6 ± 0.3	0.8 ± 0.1	1.6 ± 0.4	0.9 ± 0.2	0.7 ± 0.1	0.7 ± 0.1	0.7 ± 0.1	0.7 ± 0.1
***Ostb***	Bile acid transporter	**LFD**	1.2 ± 0.2	1.4 ± 0.2	0.8 ± 0.2	1.6 ± 0.3	1.0 ± 0.1	0.9 ± 0.1	1.0 ± 0.1	0.7 ± 0.1
		**HFD**	1.3 ± 0.2	1.0 ± 0.3	0.7 ± 0.1	1.4 ± 0.4	1.1 ± 0.1 **^c,e^**	0.6 ± 0.0	0.8 ± 0.1	0.6 ± 0.0
***Shp***	Bile acid synthesis	**LFD**	1.3 ± 0.4	0.5 ± 0.1 **^d^**	1.0 ± 0.2	0.4 ± 0.1	1.0 ± 0.1	1.0 ± 0.2	0.8 ± 0.1	0.8 ± 0.1
		**HFD**	1.0 ± 0.3 **^c^**	4.8 ± 2.2 **^e^**	1.5 ± 0.4	1.4 ± 0.2	1.3 ± 0.2 **^c,e^**	0.7 ± 0.1	0.9 ± 0.1	0.9 ± 0.2

## Data Availability

The original contributions presented in this study are included in the article. Further inquiries can be directed to the corresponding author.

## Supplementary Materials

The following supporting information can be downloaded at: https://www.mdpi.com/article/10.3390/toxics13080639/s1. Table S1: List of primers for qPCR. Table S2: List of primers for qPCR ordered from Bio-Rad. Tables S3: Significance values for statistics of hypothalamic gene expression. Blank cell indicates there is no significant value. Gray box indicates gene expression was not conducted. Table S4: Significance values for statistics of hepatic gene expression. Blank cell indicates there is no significant value. Table S5: Significance values for statistics of Figure 2, Figure 3, Figure 4, Figure 5, Figure 6 and Figure 7. Blank cell indicates there is no significant value. Table S6: Significance values for statistics of behavior tests. Blank cell indicates there is no significant value. Figure S1: Open field test- (A) distance traveled (m), (B) mean speed (m/s), and (C) perimeter visits. Elevated plus maze- (D) distance traveled (m), (E) mean speed (m/s), and (F) distance traveled in closed-arm (m). Light/dark box emergence test- (G) distance traveled (m), (H) mean speed (m/s), and (I) light zone exits. Data are represented as mean ± SEM and dots represent the sample size (number of litters) per treatments per sex (* = *p* < 0.05, ** = *p* < 0.01, **** = *p* < 0.0001).

## Abbreviations

The following abbreviations are used in this manuscript:

AGD	Anogenital Distance
AUC	Area Under the Curve
BDNF	Brain-Derived Neurotrophic Factor
BDCPP	Bis(1,3-dichloro-2-propyl)phosphate
BMI	Body Mass Index
CANDLE	Conditions Affecting Neurocognitive Development and Learning in Early Childhood
CLAMS	Comprehensive Lab Animal Monitoring System
DBUP	Di-isobutyl Phosphate
DOHaD	Developmental Origins of Health and Disease
DIBP	Di-isobutyl Phthalate
DIO	Diet-Induced Obesity
EPM	Elevated Plus Maze
ERα	Estrogen Receptor Alpha
ERE	Estrogen Response Element
GD	Gestational Day
GTT	Glucose Tolerance Test
HFD	High-Fat Diet
ITT	Insulin Tolerance Test
LDB	Light-Dark Box
LFD	Low-Fat Diet
MBH	Mediobasal Hypothalamus
NHANES	National Health and Nutrition Examination Survey
OFT	Open Field Test
OPE	Organophosphate Ester
OPFR	Organophosphate Flame Retardants
PBDE	Polybrominated Diphenyl Ether
PND	Postnatal Day
PPARγ	Peroxisome Proliferator-Activated Receptor Gamma
RAR	Retinoic Acid Receptor
RER	Respiratory Exchange Ratio
TCP	Tricresyl Phosphate
TDCPP	Tris(1,3-dichloro-2-propyl) Phosphate
TPP	Triphenyl Phosphate
