# Supplementary material for: Dual Effects of Maternal Diet and Perinatal Organophosphate Flame Retardant Treatment on Offspring Development, Behavior and Metabolism
Source: Toxics. 2025 Jul 29;13(8):639. doi: 10.3390/toxics13080639 (PMC12390071; doi:10.3390/toxics13080639)
Supplement: Supplementary file 1 [file toxics-13-00639-s001.zip › toxics-3672742-supplementary.pdf]

**Supplemental Figures**

**Table S1**. List of primers for qPCR.

| Gene | Accession # | Forward Primer | Reverse Primer |
|---|---|---|---|
| Actb | NM_007393.3 | GCCCTGAGGCTCTTTTCCA | TAGTTTCATGGATGCCACAGGA |
| Agrp | NM_007427.2 | CTCCACTGAAGGGCATCAGAA | ATCTAGCACCTCCGCCAAA |
| Bdnf | NM_001316310.1 | TCATACTTCGGTTGCATGAAGG | AGACCTCTCGAACCTGCCC |
| Bsep | NM_021022.3 | CTGCCAAGGATGCTAATGCA | CGATGGCTACCCTTTGCTTCT |
| Cart | NM_013732 | GCTCAAGAGTAAACGCATTCC | GTCCCTTCACAAGCACTTCAA |
| Cd36 | NM_001159558.1 | GATGACGTGGCAAAGAACAG | TCCTCGGGGTCCTGAGTTAT |
| Claudin-5 | NM_013805.4 | GTTAAGGCACGGGTAGCACT | TACTTCTGTGACACCGGCAC |
| Cyp2b10 | NM_009999.4 | GACTTTGGGATGGGAAAGAG | CCAAACACAATGGAGCAGAT |
| Cyp3a11 | NM_007818.3 | ACAAACAAGCAGGGATGGAC | GGTAGAGGAGCACCAAGCTG |
| Cyp4a10 | NM_010011.3 | CACACCCTGATCACCAACAG | TCCTTGATGCACATTGTGGT |
| Cyp7a1 | NM_007824.2 | AACAACCTGCCAGTACTAGATAGC | GTGTAGAGTGAAGTCCTCCTTAGC |
| Dgat2 | NM_026384.3 | ACTCTGGAGGTTGGCACCAT | GGGTGTGGCTCAGGAGGAT |
| Esr1 | NM_007956 | GCGCAAGTGTTACGAAGTG | TTCGGCCTTCCAAGTCATC |
| Fasn | NM_007988.3 | GGGTTCTAGCCAGCAGAGTC | TCAGCCACTTGAGTGTCCTC |
| Foxo1 | NM_019739 | CAATGGCTATGGTAGGATGG | TTTAAATGTAGCCTGCTCAC |
| G6pc | NM_013732 | TGCACCGCAAGAGCATT | GCCTCCTGTCGGATACAGAA |
| Gapdh | NM_008084.2 | TGACGTGCCGCCTGGAGAAA | AGTGTAGCCCAAGATGCCCTTCAG |
| Ghsr | NM_177330 | CAGGGACCAGAACCACAAAC | AGCCAGGCTCGAAAGACT |
| Hprt | NM_013556 | GCTTGCTGGTGAAAAGGACCTCTCGAAG | CCCTGAAGTACTCATTATAGTCAAGGGCAT |
| Insr | NM_010568 | GTGTTCGGAACCTGATGAC | GTGATACCAGAGCATAGGAG |
| Kiss1 | NM_178260 | TGATCTCAATGGCTTCTTGGCAGC | CTCTCTGCATACCGCGATTCCTTT |
| Lepr | NM_146146.2 | AGAATGACGCAGGGCTGTAT | TCCTTGTGCCCAGGAACAAT |
| Npy | NM_023456 | ACTGACCCTCGCTCTATCTC | TCTCAGGGCTGGATCTCTTG |
| Ostb | NM_178933 | GCAGCTGTGGTGGTCATTAT | TAGGCTGTTGTGATCCTTGG |
| Pdyn | NM_018863 | AGCTTGCCTCCTCGTGATG | GGCACTCCAGGGAGCAAAT |
| Pepck | NM_011044.2 | AGCGGATATGGTGGGAAC | GGTCTCCACTCCTTGTTC |
| Pomc | NM_008895 | GGAAGATGCCGAGATTCTGC | TCCGTTGCCAGGAAACAC |
| Ppara | NM_011144.6 | CTAACCTTGGGCCACACCT | CGGGTAACCTCGAAGTCTGA |
| Pparg | NM_011146.3 | CTGCTCAAGTATGGTGTCCATGAG | GAGGAACTCCCTGGTCATGAATC |
| Shp | NM_011850.3 | TCTGCAGGTCGTCCGACTATTC | AGGCAGTGGCTGTGAGATGC |
| Tac2 | NM_001199971 | CGTGACATGCACGACTTC | CCAACAGGAGGACCTTAC |
| Vimentin | NM_011701.4 | CTGCGAGAGAAATTGCAGGAGG | AGGTCAAGACGTGCCAGAGAA |
| Zo1 | NM_009386.3 | GCCGCTAAGAGCACAGCA | TGGAGGTTTCCCCACTCTGA |

**Table S2**. List of primers for qPCR ordered from Bio-Rad.

| Gene | Assay ID |
|---|---|
| Claudin-3 | qMmuCED0001019 |
| Dysferlin | qMmuCID0021806 |
| Lpl1 | qMmuCED0044441 |
| Lrp2 | qMmuCID0015664 |
| Occludin | qMmuCID0005446 |
| Plvap | qMmuCID0010643 |

**Table S3**. Significance values for statistics of hypothalamic gene expression. Blank cell indicates there is no significant value. Gray box indicates gene expression was not conducted.

| | PND 0 | | | | | | | | PND 14 | | | | | | | |
|---|---|---|---|---|---|---|---|---|---|---|---|---|---|---|---|---|
| | sex | | diet | | txt | | interaction | | sex | | diet | | txt | | interaction | |
| | F value | P value | F value | P value | F value | P value | F value | P value | F value | P value | F value | P value | F value | P value | F value | P value |
| Agrp | | | | | | | | | | | F (1, 55) = 16.52 | P=0.0002 | | | | |
| Bdnf | F (1, 51) = 16.20 | P=0.0002 | | | | | sex*diet*txt (F (1, 51) = 4.923) | sex*diet*txt (P=0.0310) | F (1, 53) = 9.500 | P=0.0033 | F (1, 53) = 57.36 | P<0.0001 | | | diet*txt (F (1, 53) = 4.421) | diet*txt (P=0.0403) |
| Cart | | | F (1, 53) = 6.553 | P=0.0134 | | | | | | | F (1, 55) = 31.29 | P<0.0001 | | | | |
| Esr1 | F (1, 52) = 14.45 | P=0.0004 | | | | | | | F (1, 56) = 12.85 | P=0.0007 | | | F (1, 56) = 11.46 | P=0.0013 | sex*txt (F (1, 56) = 10.70); diet*txt (F (1, 56) = 4.279); sex*diet*txt (F (1, 56) = 6.829) | sex*txt (P=0.0018); diet*txt (P=0.0432); sex*diet*txt (P=0.0115) |
| Foxo1 | | | | | F (1, 50) = 5.950 | P=0.0183 | sex*diet*txt (F (1, 50) = 4.613) | sex*diet*txt (P=0.0366) | | | F (1, 55) = 52.14 | P<0.0001 | | | | |
| Ghsr | F (1, 52) = 6.792 | P=0.0119 | F (1, 52) = 11.08 | P=0.0016 | | | diet*txt (F (1, 52) = 4.349) | diet*txt (P=0.0419) | | | | | F (1, 56) = 6.561 | P=0.0131 | | |
| Insr | F (1, 52) = 6.008 | P=0.0176 | | | F (1, 52) = 81.48 | P<0.0001 | | | F (1, 56) = 11.52 | P=0.0013 | | | | | sex*txt (F (1, 56) = 13.90); diet*txt (F (1, 56) = 4.290) | sex*txt (P=0.0005); diet*txt (P=0.0430) |

| | | | | | | | | | | | | | | | | |
|---|---|---|---|---|---|---|---|---|---|---|---|---|---|---|---|---|
| Kiss1 | F (1, 51) = 158.0 | P<0.0001 | F (1, 51) = 22.99 | P<0.0001 | F (1, 51) = 60.38 | P<0.0001 | sex*diet (F (1, 51) = 17.03); sex*txt (F (1, 51) = 32.92); diet*txt (F (1, 51) = 12.33); sex*diet*txt (F (1, 51) = 11.49) | sex*diet (P=0.0001); sex*txt (P<0.0001); diet*txt (P=0.0009); sex*diet*txt (P=0.0014) | F (1, 56) = 4.717 | P=0.0341 | | | F (1, 56) = 19.00 | P<0.0001 | | |
| Lepr | F (1, 51) = 6.659 | P=0.0128 | | | | | | | F (1, 55) = 5.408 | P=0.0238 | F (1, 55) = 10.50 | P=0.0020 | F (1, 55) = 20.41 | P<0.0001 | | |
| Npy | | | F (1, 52) = 6.444 | P=0.0142 | | | | | | | | | F (1, 53) = 7.367 | P=0.0089 | sex*diet (F (1, 53) = 9.281); diet*txt (F (1, 53) = 7.167) | sex*diet (P=0.0036); diet*txt (P=0.0099) |
| Pdyn | | | F (1, 52) = 7.487 | P=0.0085 | | | | | | | F (1, 56) = 4.702 | P=0.0344 | | | | |
| Pomc | F (1, 52) = 4.414 | P=0.0405 | | | | | sex*diet (F (1, 52) = 5.089) | sex*diet (P=0.0283) | | | | | F (1, 56) = 23.65 | P<0.0001 | | |
| Pparg | | | | | | | | | F (1, 52) = 6.198 | P=0.0160 | | | F (1, 52) = 9.114 | P=0.0039 | sex*txt (F (1, 52) = 4.506) | sex*txt (P=0.0386) |
| Tac2 | F (1, 53) = 103.1 | P<0.0001 | | | F (1, 53) = 28.54 | P<0.0001 | sex*txt (F (1, 53) = 12.55) | sex*txt (P=0.0008) | | | | | F (1, 56) = 14.50 | P=0.0003 | | |
| Claudin-3 | | | F (1, 48) = 32.24 | P<0.0001 | | | | | | | | | | | | |

| | | | | | | | | | |
|---|---|---|---|---|---|---|---|---|---|
| Claudin-5 | | | F (1, 52) = 70.03 | P<0.0001 | | | | | |
| Dysferlin | | | F (1, 47) = 7.430 | P=0.0090 | | | | | |
| Lpl1 | F (1, 53) = 7.819 | P=0.0072 | F (1, 53) = 13.57 | P=0.0005 | | | diet*txt (F (1, 53) = 6.199) | diet*txt (P=0.0160) | |
| Lrp2 | | | F (1, 53) = 52.02 | P<0.0001 | | | | | |
| Occludin | | | F (1, 50) = 89.78 | P<0.0001 | F (1, 50) = 4.449 | P=0.0400 | | | |
| Plvap | F (1, 49) = 4.304 | P=0.0433 | F (1, 49) = 11.88 | P=0.0012 | | | sex*diet*txt (F (1, 49) = 4.576) | sex*diet*txt (P=0.0374) | |
| Vimentin | F (1, 52) = 18.54 | P<0.0001 | F (1, 52) = 15.76 | P=0.0002 | | | sex*diet (F (1, 52) = 13.23); sex*diet*txt (F (1, 52) = 9.266) | sex*diet (P=0.0006); sex*diet*txt (P=0.0037) | |
| Zo1 | F (1, 53) = 17.69 | P=0.0001 | | | | | | | |

**Table S4**. Significance values for statistics of hepatic gene expression. Blank cell indicates there is no significant value.

| | PND 0 | | | | | | | | PND 14 | | | | | | | |
|---|---|---|---|---|---|---|---|---|---|---|---|---|---|---|---|---|
| | sex | | diet | | txt | | interaction | | sex | | diet | | txt | | interaction | |
| | F value | P value | F value | P value | F value | P value | F value | P value | F value | P value | F value | P value | F value | P value | F value | P value |
| Bsep | | | F (1, 39) = 6.942 | P=0.0120 | | | | | | | | | | | | |
| Cd36 | | | | | | | sex*diet (F (1, 38) = 5.066) | sex*diet (P=0.0303) | | | | | | | | |
| Cyp2b10 | | | F (1, 38) = 5.519 | P=0.0241 | F (1, 38) = 7.893 | P=0.0078 | diet*txt (F (1, 38) = 4.421) | diet*txt (P=0.0422) | | | | | | | | |
| Cyp3a11 | | | | | F (1, 39) = 5.408 | P=0.0253 | sex*diet (F (1, 39) = 4.832); sex*diet*txt (F (1, 39) = 6.188) | sex*diet (P=0.0339); sex*diet*txt (P=0.0172) | | | | | F (1, 38) = 9.423 | P=0.0039 | | |
| Cyp4a10 | | | F (1, 39) = 13.92 | P=0.0006 | | | | | | | | | F (1, 39) = 12.17 | P=0.0012 | sex*diet*txt (F (1, 39) = 6.7710) | sex*diet*txt (P=0.0130) |
| Cyp7a1 | | | F (1, 40) = 4.440 | P=0.0414 | | | | | | | | | F (1, 38) = 5.417 | P=0.0254 | | |
| Dgat2 | | | | | | | | | F (1, 39) = 4.462 | P=0.0411 | | | | | | |
| Esr1 | | | | | F (1, 39) = 15.89 | P=0.0003 | | | | | | | | | | |
| Fasn | | | | | F (1, 37) = 5.075 | P=0.0303 | | | | | F (1, 37) = 4.149 | P=0.0489 | | | | |
| Foxo1 | | | | | | | | | | | | | | | | |
| G6pc | | | | | | | sex*diet*txt (F (1, 39) = 4.516) | sex*diet*txt (P=0.0400) | | | | | | | | |

| | | | | | | | | | | | | | | | | |
|---|---|---|---|---|---|---|---|---|---|---|---|---|---|---|---|---|
| Insr | | | F (1, 40) = 5.046 | P=0.0303 | | | sex*diet (F (1, 40) = 6.482) | sex*diet (P=0.01490) | | | | | | | | |
| Lepr | | | F (1, 37) = 7.285 | P=0.0104 | F (1, 37) = 7.534 | P=0.0093 | sex*diet (F (1, 37) = 16.89); diet*txt (F (1, 37) = 14.94); sex*diet*txt (F (1, 37) = 7.158) | sex*diet (P=0.0002); diet*txt (P=0.0004); sex*diet*txt (P=0.0111) | | | | | F (1, 37) = 8.855 | P=0.0051 | | |
| Ostb | | | | | | | sex*diet (F (1, 39) = 4.746) | sex*diet (P=0.0355) | | | F (1, 39) = 12.34 | P=0.0011 | | | | |
| Pepck | | | | | | | sex*diet (F (1, 38) = 4.225) | sex*diet (P=0.0468) | | | | | | | | |
| Ppara | | | F (1, 40) = 6.216 | P=0.0169 | | | | | | | | | | | | |
| Pparg | | | | | F (1, 39) = 4.112 | P=0.0494 | sex*diet (F (1, 39) = 7.660) | sex*diet (P=0.0086) | | | | | F (1, 37) = 12.13 | P=0.0013 | | |
| Shp | | | | | F (1, 38) = 4.896 | P=0.0330 | diet*txt (F (1, 38) = 4.268) | diet*txt (P=0.0457) | | | | | | | | |

**Table S5**. Significance values for statistics of Figures 2-7. Blank cell indicates there is no significant value.

| | sex (or time**) | | diet | | txt | | interaction | |
|---|---|---|---|---|---|---|---|---|
| | F value | P value | F value | P value | F value | P value | F value | P value |
| **PND 7 AGD** | | | | | | | | |
| Distance (mm) | F (1, 86) = 590.3 | P<0.0001 | F (1, 86) = 28.97 | P<0.0001 | | | sex*txt (F(1,86)=4.564); txt*diet (F(1,86)=9.530) | sex*txt (P=0.0355); txt*diet (P=0.0027) |
| **Body Weight/Composition** | | | | | | | | |
| Body Weight (g) | F (1, 73) = 206.6 | P<0.0001 | | | | | | |
| % Lean Mass | F (1, 72) = 44.35 | P<0.0001 | | | F (1, 72) = 9.804 | P=0.0025 | txt*diet (F(1,72)=4.069) | txt*diet (P=0.0474) |
| % Fat Mass | F (1, 74) = 19.75 | P<0.0001 | | | F (1, 74) = 4.873 | P=0.0304 | | |
| **Metabolic Phenotyping** | | | | | | | | |
| V.O2 (ml/min/kg) | F (1, 73) = 109.8 | P<0.0001 | | | F (1, 73) = 10.52 | P=0.0018 | | |
| V.CO2 (ml/min/kg) | F (1, 73) = 77.16 | P<0.0001 | | | F (1, 73) = 34.69 | P<0.0001 | | |
| RER (V.CO2/V.O2) | | | | | F (1, 71) = 79.86 | P<0.0001 | txt*diet (F(1,71)=4.756 | txt*diet (P=0.0325) |
| Heat (kCal/hr) | | | | | F (1, 73) = 23.47 | P<0.0001 | | |
| Wheel Running Counts | F (1, 64) = 60.28 | P<0.0001 | | | F (1, 64) = 30.48 | P<0.0001 | sex*txt*diet (F(1,64)=4.235 | sex*txt*diet (P=0.0437) |
| Female Wheel Running** | F (23, 789) = 109.1 | P<0.0001 | | | F (1, 789) = 138.6 | P<0.0001 | time*txt (F(23,789)=6.636); txt*diet (F(1,789)=28.46); time*txt*diet (F(23,789)=3.202) | time*txt (P<0.0001); txt*diet (P<0.0001); time*txt*diet (P<0.0001) |
| **Tolerance Tests** | | | | | | | | |
| Male GTT glucose (mg/dL)** | F (5, 230) = 97.84 | P<0.0001 | | | F (1, 230) = 4.526 | P=0.0344 | | |

| | | | | | | | | |
|---|---|---|---|---|---|---|---|---|
| Female GTT glucose (mg/dL)** | F (5, 210) = 202.4 | P<0.0001 | F (1, 210) = 4.119 | P=0.0437 | | | txt*diet<br>(F(1,210)=5.773) | txt*diet<br>(P=0.0171) |
| AUC GTT (mg/dL/min) | F (1, 74) = 16.27 | P=0.0001 | | | | | | |
| Male ITT glucose (mg/dL)** | F (5, 225) = 81.60 | P<0.0001 | | | F (1, 225) = 42.25 | P<0.0001 | time*diet<br>(F(5,225)=5.726) | time*diet<br>(P<0.0001) |
| Female ITT glucose (mg/dL)** | F (5, 194) = 63.54 | P<0.0001 | | | | | time*diet<br>(F (5,194)=3.330) | time*diet<br>(P=0.0066) |
| AUC ITT (mg/dL/min) | F (1, 59) = 23.41 | P<0.0001 | | | F (1, 59) = 16.33 | P=0.0002 | | |

**Table S6**. Significance values for statistics of behavior tests. Blank cell indicates there is no significant value.

| | sex | | diet | | txt | | interaction | |
|---|---|---|---|---|---|---|---|---|
| | F value | P value | F value | P value | F value | P value | F value | P value |
| **Open Field Test** | | | | | | | | |
| Distance (m) | | | | | F (1, 69) = 8.663 | P=0.0044 | | |
| Speed (m/s) | | | | | F (1, 69) = 8.732 | P=0.0043 | | |
| Perimeter Visits | F (1, 67) = 7.014 | P=0.0101 | F (1, 67) = 5.639 | P=0.0204 | | | | |
| Latency to 1st Entry (20 cm) | | | | | | | txt*diet (F(1, 65)=6.087) | txt*diet (P=0.0163) |
| % Time in 10 cm Center | | | | | F (1, 66) = 12.26 | P=0.0008 | txt*diet (F(1, 66)=4.579) | txt*diet (P=0.0361) |
| **Elevated Plus Maze** | | | | | | | | |
| Distance (m) | | | | | F (1, 73) = 15.76 | P=0.0002 | | |
| Speed (m/s) | | | | | F (1, 73) = 15.49 | P=0.0002 | | |
| Closed-Arm Distance (m) | | | | | F (1, 72) = 30.35 | P<0.0001 | | |
| Closed-Arm Entries | | | | | F (1, 74) = 27.72 | P<0.0001 | | |
| **Light Dark Box** | | | | | | | | |
| Distance (m) | | | | | F (1, 73) = 9.463 | P=0.0030 | | |
| Speed (m/s) | | | | | F (1, 73) = 9.966 | P=0.0023 | | |
| Light Zone Exits | | | | | F (1, 71) = 10.57 | P=0.0018 | | |
| Light Zone Entries | | | | | F (1, 70) = 11.67 | P=0.0011 | | |

| | | | | | | | | |
|---|---|---|---|---|---|---|---|---|
| **Y-Maze** | | | | | | | | |
| Distance (m) | | | | | | | | |
| Speed (m/s) | | | | | | | | |
| % Time in Unknown Arm | | | | | F (1, 73) = 4.015 | P=0.0488 | sex*txt (F(1,73)=4.586) | sex*txt (P=0.0356) |

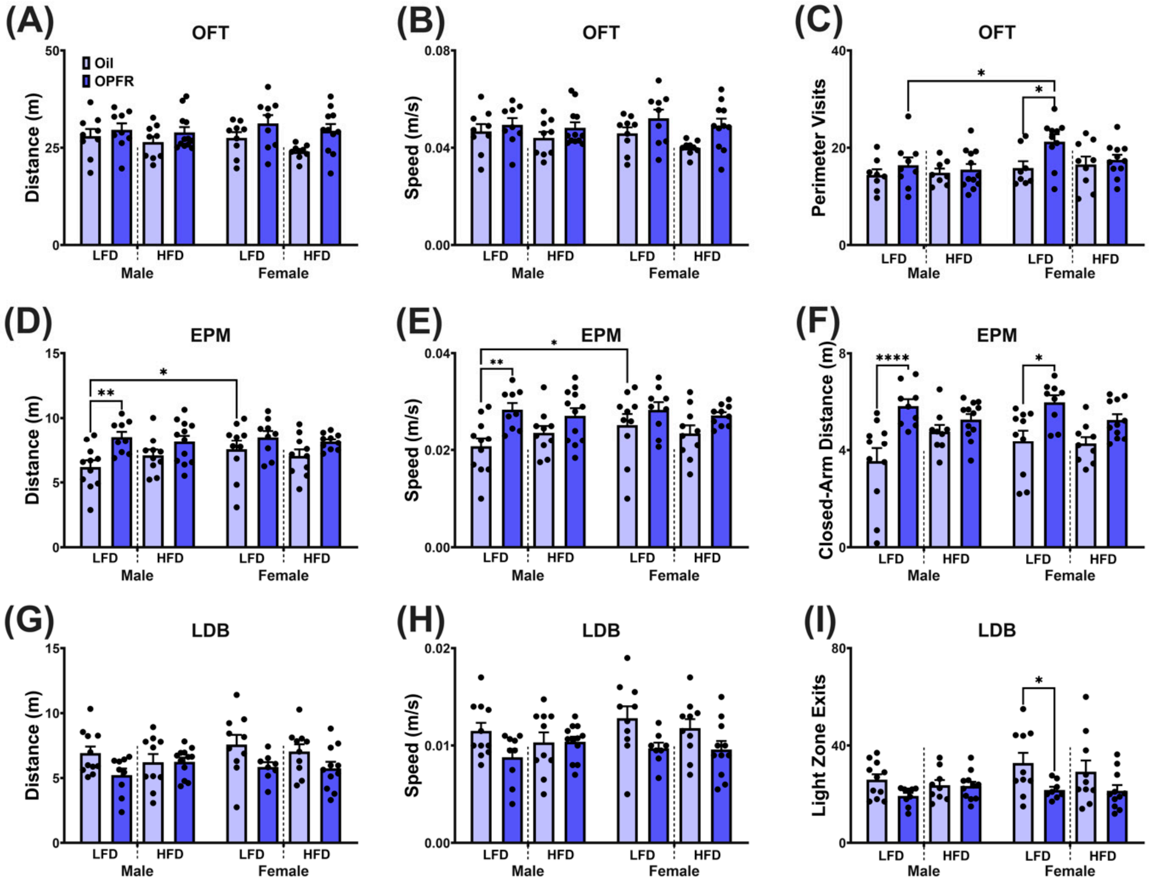


**Figure S1**. Open field test- (**A**) distance traveled (m), (**B**) mean speed (m/s), and (**C**) perimeter visits. Elevated plus maze- (**D**) distance traveled (m), (**E**) mean speed (m/s), and (**F**) distance traveled in closed-arm (m). Light/dark box emergence test- (**G**) distance traveled (m), (**H**) mean speed (m/s), and (**I**) light zone exits. Data are represented as mean ± SEM and dots represent the sample size (number of litters) per treatments per sex (*= P<0.05, **= P<0.01, ****= P<0.0001).
